# Attachment- and Emotion-Focused Parenting Interventions for Child and Adolescent Externalizing and Internalizing Behaviors: A Meta-Analysis

**DOI:** 10.1007/s10567-022-00401-8

**Published:** 2022-06-10

**Authors:** Samantha Jugovac, Richard O’Kearney, David J. Hawes, Dave S. Pasalich

**Affiliations:** 1 Research School of Psychology, Australian National University, Canberra, Australia; 2 School of Psychology, The University of Sydney, Sydney, Australia

**Keywords:** Attachment theory, Behavior problems, Emotion socialization, Meta-analysis, Parent–child relationship, Parenting intervention

## Abstract

Attachment- and emotion-focused parenting interventions (AE) have grown in popularity as an alternative to behavioral parent training (BPT) for children and adolescents. AE go beneath behavior by helping parents understand and respond to their child’s underlying attachment and emotional needs. Past reviews have examined their effects on attachment security and caregiver sensitivity, though less is known regarding their effects on child mental health symptoms. Reported here is the first systematic review and meta-analysis of individual and group AE on externalizing behavior (EXT) and internalizing behavior (INT) for children aged 0–18 years. A search of four databases prior to July 2021 elicited 43 studies that met eligibility criteria. Meta-analysis revealed that AE were superior to waitlist controls for EXT (*SMD* = − 0.17) and INT (*SMD* = − 0.34). Effects were sustained at follow-up periods of 6 months and greater, and AE considered to target child mental health were significantly more effective than those that did not in reducing EXT and INT. Two studies retrieved directly compared AE to BPT, which showed no evidence of a difference for follow-up measures of EXT. No studies compared AE to BPT on INT. AE demonstrated no evidence of superiority compared to controls for parent mental health. Findings support the potential for AE to reduce EXT and INT in children and adolescents; however, future research should consider the relative effectiveness of AE.

**Supplementary Information:**

The online version contains supplementary material available at 10.1007/s10567-022-00401-8.

Left untreated, child externalizing behaviors (EXT) (e.g., noncompliance, aggression) and internalizing behaviors (INT) (e.g., anxiety, depression) can lead to significant adverse outcomes across development, including later mental health difficulties, reduced academic achievement, and unemployment (Clark et al., 2017; Goodman et al., 2011; Kim-Cohen et al., 2003). The quality of parenting and parent–child interactions are key risk factors linked to the development and maintenance of these problem behaviors and are modifiable through intervention. For example, reduced parental warmth and sensitivity are associated with both increased EXT (Hoeve et al., 2009) and INT (McLeod et al., 2007a, 2007b). In this light, much research has focused on examining the effects of parenting interventions on child and adolescent EXT and INT.

In the literature, parenting interventions are typically categorized as “behavioral” or “nonbehavioral.” Accordingly, we first review evidence on behavioral parent training (BPT), and then review the growing literature on nonbehavioral parenting interventions, with an emphasis on the subcategory of attachment- and emotion-focused parenting interventions (AE). The latter provides the focus of this review, which aims to investigate the effectiveness of individual and group AE on child and adolescent EXT and INT compared to waitlist controls, BPT, and other active comparators.

## Behavioral Parenting Interventions

BPT has been the predominant model of parenting interventions since the 1960s. BPT derived from social learning theory, which stipulates that child behaviors are strengthened and weakened through parent reinforcers (Scott & Dadds, 2009). EXT are thought to be maintained through coercive parent–child reinforcement traps, whereby a child and parent engages in aversive behaviors (e.g., yelling) until either person capitulates, ultimately reinforcing the other’s aversive behavior (Patterson, 1982). BPT aims to break coercive cycles by shifting parents’ attention from children’s problematic behaviors to their desirable behaviors through strategies such as praise, clear instructions, logical consequences, planned ignoring, and timeout from positive reinforcement (McMahon & Pasalich, 2020). Examples of well-researched BPT programs include the following: Helping the Noncompliant Child (HNC, Forehand & McMahon, 1981), The Incredible Years (IY, Webster-Stratton, 2005), and Triple P-Positive Parenting Program (Sanders, 1999). BPT is considered the gold-standard for treating EXT, with meta-analytic findings demonstrating its effectiveness across childhood (Kaminski & Claussen, 2017; Mingebach et al., 2018). While BPT was not originally designed to directly target INT, findings suggest that it may still be effective at reducing INT for children and adolescents (Forehand et al., 2013; Zarakoviti et al., 2021).

Although BPT effectively reduces EXT and to a lesser extent, decreases INT, approximately 25–33% of children do not appear to benefit from these programs (Scott & Dadds, 2009), with high dropout and low attendance rates reported. For example, a review of BPT found that 25% of eligible participants did not begin treatment, and 26% dropped out before completing treatment (Chacko et al., 2016). Research is limited on variables that may influence attrition in BPT programs, or produce barriers to positive treatment outcomes. Notwithstanding this, past findings suggest that baseline variables such as socioeconomic status, parent mental health, family history of trauma or attachment difficulties, and parent attributions may moderate the effect of BPT outcomes for families (Assenany & McIntosh, 2002; Havighurst et al., 2020; Maliken & Katz, 2013; Scott & Dadds, 2009). Notably, parent mental health problems have been shown to be a moderator of BPT outcomes (Maliken & Katz, 2013). Accordingly, researchers have highlighted the need to focus on parents’ understanding and management of their own emotions in addition to their child’s (Havighurst et al., 2020), which is not a key focus in some BPT programs.

Another potential barrier to participation in or positive treatment outcomes for BPT includes preference for an alternative theoretical model of intervention. Research in the general field of evidence-based practice with health professionals, including clinical psychologists, demonstrates that practitioners’ preferred theoretical orientation is a significant barrier to implementation of evidence-based treatments (Lilienfeld et al., 2013; Pagato et al., 2007). With regard to BPT, the use of timeout—i.e., a core component of BPT, whereby a child is placed in a safe, neutral space for a brief period of time away from parent attention (Kaminski et al., 2008)—has been the source of ongoing debate among practitioners and researchers, with many parents and professionals re-evaluating its strengths and challenges [see Dadds and Tully (2019) for a recent review]. Moreover, a recent community-based study exploring why some parents prefer not to use timeout found that they favor alternative parenting strategies believed to value connection, “attachment,” and co-regulation in the parent–child relationship (Canning et al., 2021). In summary, although BPT has a substantial evidence base and is widely disseminated, there is a timely need to investigate the collective evidence for alternative models of parenting interventions targeting EXT and INT that may be better suited to parents who prefer and value relational and emotion-oriented approaches to parenting.

## Attachment- and Emotion-Focused Parenting Interventions

The most researched “nonbehavioral” parenting interventions involve programs based on (1) attachment theory and (2) emotion socialization theory, which are characterized by a focus on affective processes in the parent–child relationship and understanding the meaning of, and/or emotional needs underlying, child behavior. According to attachment theory, a secure attachment develops when a caregiver sensitively responds to their child’s needs and the child can alleviate their distress by seeking proximity to the caregiver (Ainsworth et al., 1978; Bowlby, 1982). By contrast, when a caregiver inconsistently responds to their child’s needs, the child may develop an ambivalent attachment, which is characterized by an approach and resistance pattern of interaction with the caregiver when distressed. Children whose caregivers are consistently unresponsive to or rejecting of their needs are at risk of developing an avoidant attachment wherein they do not seek proximity to their caregiver when distressed (Ainsworth et al., 1978). Of most concern for the development of psychopathology, a disorganized attachment is evident in children who do not develop a coherent strategy to assuage their distress and is linked to fearful and frightening caregiving (Lyons-Ruth & Spielman, 2004).

Attachment-based interventions such as Attachment- and Bio-behavioral Catch-up (Dozier et al., 2011) and Circle of Security (Hoffman et al., 2006) focus on improving attachment security through targeting caregiving behaviors. These include caregiver sensitivity (i.e., caregivers’ ability to notice and determine their child’s needs) and reflective functioning (i.e., caregivers’ capacity to understand their own and their child’s mental states including feelings and beliefs) (Kobak et al., 2015). Meta-analytic evidence supports associations between insecure attachment styles and EXT and INT. In particular, disorganized attachment most strongly predicts EXT and avoidant attachment is most strongly associated with INT (Groh et al., 2017). In addition to attachment styles, caregiving behaviors including caregiver sensitivity and reflective functioning are associated with both EXT and INT (Carlone & Milan, 2020; Dejko-Wanczyk et al., 2020; Kok et al., 2013; Wang et al., 2013). Given the established links between quality of caregiving and attachment, attachment-based interventions have predominantly focused on evaluating changes in caregiver sensitivity and attachment security (e.g., Bakermans-Kranenburg et al., 2003). However, surprisingly, there has been less research attention on other child outcomes such as EXT and INT, which are linked to attachment and associated caregiving behaviors.

Parenting interventions based on emotion socialization theory—e.g., Tuning in to Kids (Havighurst et al., 2010)—also focus on the parent–child relationship and aim to support parents in understanding and responding to emotional needs related to child behavior. The rationale for emotion socialization interventions is based in part on associations between greater emotional competence and less EXT and INT (Saarni, 1999). Emotional competence can be strengthened through parents’ “emotion coaching.” Emotion coaching principles include having awareness of children’s emotions and recognizing emotional moments as opportunities for teaching and intimacy, listening empathetically, validating feelings, facilitating children to label their emotions, and helping children problem-solve (Gottman & DeClaire, 1997). Parent emotion socialization behaviors including emotion coaching are significantly associated with both EXT (Johnson et al., 2017) and INT (Shortt et al., 2016; Suveg et al., 2005), and meta-analytic findings show that child emotion regulation is a protective factor for EXT and INT (Daniel et al., 2020).

Researchers have recently considered the implications of attachment theory for emotion socialization theory and vice versa, as in part, the attachment relationship develops through caregivers’ responses to child emotional cues. For instance, many of the skills within attachment-based interventions (e.g., caregiver sensitivity and reflective functioning) require parents to have capacity for emotion regulation (Hajal & Paley, 2020). Given similarities of theoretical origin and intervention targets between interventions guided by emotion socialization and attachment theory, for the purposes of this review, we combine these two interventions as AE. We define AE as those that go beneath behavior and aim to strengthen the emotional quality of the parent–child relationship by helping caregivers understand children’s attachment and emotional needs expressed through their behavior.

Emerging evidence of randomized controlled trials (RCTs) investigating the effects of individual and group AE on EXT and INT has revealed significant improvements for children and adolescents (e.g.., Baker et al., 2015; Havighurst et al., 2019; Moretti et al., 2018) and potentially comparable effects to BPT (Duncombe et al., 2016; Högström et al., 2017). Furthermore, AE may produce sleeper effects, whereby positive intervention effects may be enlarged at follow-up compared to post-treatment, hence requiring longer follow-up assessments to identify changes (Bakermans-Kranenburg et al., 2003). However, other studies have failed to find statistically significant changes in EXT or INT for AE (e.g., Adkins et al., 2021, Rolock et al., 2021; van Doesum et al., 2008). Thus, without a systematic review and meta-analysis of pooled RCTs, we cannot be certain of the effectiveness of AE on EXT and INT for children and adolescents.

## Previous Reviews of Parenting Intervention Effects on Externalizing and Internalizing Outcomes

Several prior systematic reviews and meta-analyses have investigated the effectiveness of parenting interventions broadly on both EXT and INT; however, AE appear to be frequently missing. For example, a meta-analysis of past meta-analyses examining the effectiveness of any parenting intervention for the treatment of EXT in clinic-referred children aged under 13 (Mingebach et al., 2018) found a significant moderate effect for reduced EXT (effect size = 0.46, 95% CI 0.35–0.55); though, only 2 of 26 meta-analyses included nonbehavioral interventions (Leijten et al., 2018; Lundahl et al., 2006). The first meta-analysis compared behavioral to nonbehavioral interventions on EXT but the nonbehavioral studies did not reference attachment or emotion socialization theory (Lundahl et al., 2006). The second meta-analysis examined the integration of “relationship enhancement” with BPT in a single intervention for reducing EXT; however, it did not consider the isolated effects of AE without behavioral components (Leijten et al., 2018). Although these past studies increase our understanding of BPT, the specific effects of AE on child EXT and INT remain unclear.

In addition to the predominant focus on BPT in past research, it is possible that AE were often excluded from prior reviews of parenting interventions due to participant eligibility criteria. BPT programs were specifically developed to treat EXT and secondary problem behaviors, whereas many AE were originally developed to primarily strengthen caregiving and the parent–child relationship in targeted populations, such as children exposed to maltreatment and/or in out-of-home care (Dozier et al., 2011; Slade et al., 2018). To illustrate, a systematic review of 64 RCTs of psychosocial treatments in children 12 and younger concluded that group and individual BPT demonstrated the most support for reducing EXT; yet only one emotion-focused intervention and no attachment-based interventions were included (Kaminski & Claussen, 2017). This was most likely due to the study’s inclusion criteria requiring participants to exhibit clinically elevated EXT behavior at baseline, which is not typical for AE research. Furthermore, children in out-of-home care—who are commonly targeted in AE—were also excluded from this review.

Finally, although a recent narrative review of AE research suggests promising intervention effects for child and adolescent EXT and INT (Havighurst et al., 2020), this study did not involve a meta-analysis and is limited to studies published January 2019 through June 2020. In summary, previous research examining the effects of parenting interventions on EXT and INT have not comprehensively and systematically examined the effectiveness of AE on child and adolescent EXT and INT. This is a significant gap in the literature with considerable importance for research and practice concerning parenting interventions and child wellbeing.

Thus far, we have primarily highlighted the significant gap in the literature on AE effects on child EXT and INT, though there is also limited research around its effects on parent mental health and wellbeing outcomes. Previous reviews of AE have investigated intervention-induced improvements in parent skills (e.g., caregiver sensitivity; Bakermans-Kranenburg et al., 2003) but not parent mental health, which may also be impacted by some AE (e.g., Moretti et al., 2018; Weihrauch et al., 2014), particularly given that AE predominately target parents’ behavior, thoughts, and feelings (Havighurst et al., 2020; Kobak et al., 2015; Maliken & Katz, 2013). A meta-analysis on one type of AE—Circle of Security—found a medium effect on parent depressive symptoms following intervention; however, only three studies were included (Yaholkoski et al., 2016). In light of these limitations, further research on parent mental health and wellbeing outcomes following AE is needed.

## Current Review

This study advances previous research by conducting the first meta-analysis and systematic review on the effectiveness of AE for reducing EXT and INT in children and adolescents. We aimed to address research gaps by being broad in our search of existing studies within the scope of examining AE. Specifically, all AE studies that measured an EXT and/or INT outcome were included. To understand the effects of these interventions across child and adolescent development, studies included children aged under 18. Furthermore, we aimed to compare AE to the most established parenting intervention model, BPT, in order to understand whether AE could be a suitable alternative for families presenting in community and clinical settings. To this end, the following study objectives were considered:

1. Are AE more effective than waitlist comparisons in reducing child and adolescent EXT and INT?
2. Are AE as effective as BPT in reducing child and adolescent EXT and INT?
3. Are AE more effective than any active comparison in reducing child and adolescent EXT and INT?

Given past research on parenting interventions appears to be limited in identifying variables that may impact on what intervention works best for whom, this review also aimed to explore subgroup analyses to consider whether participant characteristics (e.g., age, caregiver type) at baseline may moderate AE outcomes. The review also considered the effect of intervention (e.g., delivery format) and outcome (e.g., length of follow-up) characteristics on treatment outcomes. While research suggests AE may improve parenting skills (e.g., caregiver sensitivity; Bakermans-Kranenburg et al., 2003), less is known about other important parent outcomes. Therefore, as a secondary outcome, we aimed to explore whether AE may improve parents’ mental health and wellbeing (i.e., stress, depression, anxiety).

## Method

### Study Design and Protocol

The protocol for this systematic review and meta-analysis was prepared according to the preferred reporting items for systematic reviews and meta-analyses (PRISMA) guidelines (Moher et al., 2009). The prospectively registered protocol can be retrieved from PROSPERO (CRD42017081873).

#### Eligibility Criteria

##### Types of Studies

All studies that measured the effectiveness of AE on EXT or INT against a comparator were included. That is, all controlled trials (including quasi-randomized) were included. Cross-sectional, case series, and case report designs were not included. This approach was taken for two main reasons. Firstly, AE research is relatively recent and given this was the first meta-analysis investigating the effects of AE on EXT/INT, we were uncertain of the availability of published RCTs and wanted to capture a wider range of studies. Secondly, non-randomized studies are suggested to be more likely to reflect clinical practice, particularly when RCTs may not have been a feasible or practical option due to waitlist times and ethical considerations (Faber et al., 2016). Moreover, some AE rapidly grew in popularity in the community prior to the availability of randomized effectiveness trials (Moretti et al., 2018). Notwithstanding this, we considered the potential bias introduced through the inclusion of non-randomized studies (Reeves et al., 2021) in quality assessment.

##### Types of Participants

Participants included children under the age of 18 and their parent(s). For the purposes of this paper, parent(s) is defined as the primary caregiver(s) of the child including birth, kinship, adoptive, and foster parents. Studies where participants’ average age was above 18, and interventions aimed at pregnant mothers were excluded. Studies that included samples of children with autism spectrum disorder (ASD) and/or an intellectual disability, and parents with substance abuse disorders were also excluded. Previous published reviews have focused on these particular populations (e.g., see Tarver et al., 2019) and prior reviews on BPT have typically excluded these populations (e.g., Kaminski & Claussen, 2017; Tully & Hunt, 2016).

##### Type of Interventions

To be considered for inclusion, the intervention was required to primarily target the parent(s) of the child (i.e., > 50% of the intervention involved therapist-parent contact). At least one intervention arm was required to be an AE. That is, the intervention was required to be primarily rooted in attachment theory, informed by principles of emotion coaching or focus on teaching parents skills to link children’s emotions to behaviors (including their ability to reflect on their own and their child’s emotions). Interventions were focused on improving parents’ skills in sensitive responding, emotion coaching, and/or reflecting functioning. Each identified study was assessed by at least two reviewers (always including the lead author, S.J.) for eligibility of the intervention as AE. Each reviewer was provided with a predetermined list of interventions [see Appendix A (Online Resource 1), for example, of interventions] that was created based on expert knowledge and prior literature that has categorized these interventions previously (e.g., Steele & Steele, 2018; Troutman, 2015). Where an intervention was not on this predetermined list, the two reviewers discussed whether it should be included, and if there was no consensus, a third author (D.P.; expert in development and implementation of AEs) was consulted. All authors of this paper (who are trained and experienced in administering various parenting interventions) also reviewed and approved the final list of included interventions for eligibility as an AE. If a study included a ‘blended’ parenting intervention—an intervention that combined more than one theoretical orientation—it was excluded in order to better understand the stand-alone effect of AE. See Leijten et al. (2018) for a previous review that considered the integration of behavioral and attachment theoretical orientations. Although there may be a level of subjectivity in categorizing theoretical orientation of parenting interventions, for the purpose of this paper, we based eligibility as an AE on the original primary theoretical underpinnings of an intervention. In other words, we included interventions that originated out of attachment theory or emotion socialization theory without direct influence of social learning theory or alternate theoretical orientations.

##### Types of Outcomes

Primary outcomes included post-treatment and/or follow-up scores on a measure of EXT and/or INT. Given EXT and INT cover a broad range of symptoms, broadband measures such as the Child Behavior Checklist, which has higher order factors for EXT and INT (CBCL, Achenbach, 1994), and narrowband measures such as the Beck Depression Inventory-II (BDI-II; Beck et al., 1996), which measures a specific type of INT (i.e., depressive symptoms), were extracted. Any measuring tool or respondent was included (e.g., parent-report, self-report). Secondary outcomes included post-treatment and/or follow-up measures of parent mental health and wellbeing (i.e., stress, anxiety, depression).

#### Search Strategy

Studies were primarily sourced through electronic databases: Cochrane, Scopus, PsychInfo, and PubMed. See Appendix B (Online Resource 1) for search terms used in databases. Non-English studies were excluded. The first author also scanned reference lists of included studies and relevant published reviews. Studies were included if they were published prior to July 2021. No other limits were placed on the search including no minimum publication date.

### Data Collection

#### Selection of Studies

Two reviewers independently searched electronic databases for relevant titles and abstracts, reviewed full-text articles, and extracted data using a structured electronic data form. At each stage, any disagreements were resolved through discussion between two reviewers, and where needed, a third expert reviewer was consulted. If further data were required, the author of the paper was contacted via email. The first author only re-conducted the electronic search prior to submitting the journal for publication.

#### Data Extraction and Management

The following data were extracted from full-text articles: eligibility details, study design, participant characteristics (e.g., child age, parent age, and ethnicity), intervention details (e.g., theoretical orientation, attrition details, facilitator experience, and integrity), control details, outcome details (e.g., measure, informant, and length of follow-up), drop-outs, and missing participants. For meta-analysis, means, standard deviations, and sample sizes were collected for intervention and comparison groups at all points of data collection available following treatment. Where there were multiple papers with the same sample, typically the most comprehensive paper was used, though if several papers covered the same sample and assessed different types of outcomes (i.e., one had EXT only and one had INT only), both papers were used to extract data. However, only one outcome variable from the study sample was used in any single meta-analysis. Where possible, broadband measures of EXT or INT were used in meta-analysis; however, if a study only included a narrowband measure, this was used. If multiple different measures were available for the same outcome, one measure was chosen to be included in the meta-analysis based on the consensus of two reviewers. Given parent-report was expected to be the predominant informant, this was typically given priority to reduce heterogeneity across studies. In conjunction with this, reviewers considered inclusion of measures for the meta-analysis based on higher reliability and validity.

#### Risk of Bias Assessment

Risk of bias for individual studies was independently assessed by two reviewers using the Cochrane Risk of Bias Tool (Higgens et al., 2011). This included selection bias, performance bias, detection bias, attrition bias, reporting bias, intervention fidelity, and other bias (i.e., bias within the study that did not fit appropriately into another category). For this study, we were most interested in fidelity of the intervention and attrition bias. Given the nature of psychological intervention outcome studies, blinding of participants and personnel was not given as much weight as other characteristics (Munder & Barth, 2017). Non-randomized studies are likely to be considered ‘high’ risk of bias as there would be expected baseline group differences; these were removed in sensitivity analyses to monitor any bias on pooled effect sizes.

### Analysis and Data Synthesis

Data synthesis was conducted using Review Manager, Version 5.3 (The Cochrane Collection, 2014). The estimated intervention effect in each study was calculated using standardized mean difference (*SMD*), otherwise known as Hedges’ *g*, at post or follow-up. This method was chosen as an alternative to pre-post effect sizes in order to provide unbiased outcomes and control for other factors that may account for intervention effects (Cuijpers et al., 2017). For this paper, a *SMD* of 0.2 is considered a small effect, 0.5 is considered a moderate effect, and 0.8 is considered a large effect (Cohen 1988, as cited in Schünemann et al., 2019).

Pooling was considered for all research questions where there were three or more studies available, and heterogeneity was low to moderate. Heterogeneity was assessed using the I-squared (*I*^2^) and chi-squared (*χ*^2^) statistics. Heterogeneity was considered too high if there was a significant *χ*^2^ (*p* < 0.05) and *I*^2^ value > 59% (Higgens et al., 2021). When heterogeneity was considered low to moderate, random effects were used to pool data in a meta-analysis as it was assumed that the true effect estimate would vary for each study. If heterogeneity was too high or there were too few studies, no pooling was conducted, and individual study results were reported. Further, if individual studies did not include the necessary statistics for EXT or INT (i.e., *M*, *SD/SE*, and *N*) for each intervention arm, they were excluded from meta-analysis.

#### Subgroup and Sensitivity Analysis

In addition to the main research questions, additional subgroup and sensitivity analyses were conducted. Planned subgroup analysis included child age (infant/toddler; school-age, adolescence), caregiver type (birth, adoptive, foster, kinship), length of follow-up (post-treatment, 6-month and greater), and clinical levels of EXT and/or INT at baseline (elevated versus not elevated). Sensitivity analyses were conducted to examine the impact of individual study risk of bias by removing high-risk studies, and the impact of heterogeneity in results by removing clear outliers (Ryan, 2016). Outliers were observed through visual inspection of funnel plots and/or forest plots and then excluded in a sensitivity analysis if the confidence interval (CI) of the study did not overlap with the pooled effect size. That is, the upper CI of the individual study was lower than the lower bound of the pooled effect size CI (Viechtbauer & Cheung, 2010).

#### Publication Bias

For each meta-analysis conducted with at least 10 studies, publication bias was assessed through funnel plots, charted by effect size. Higgens et al. (2021) advise against the use of funnel plots for meta-analyses under 10 studies.

## Results

### Study Characteristics

**Fig. 1:**
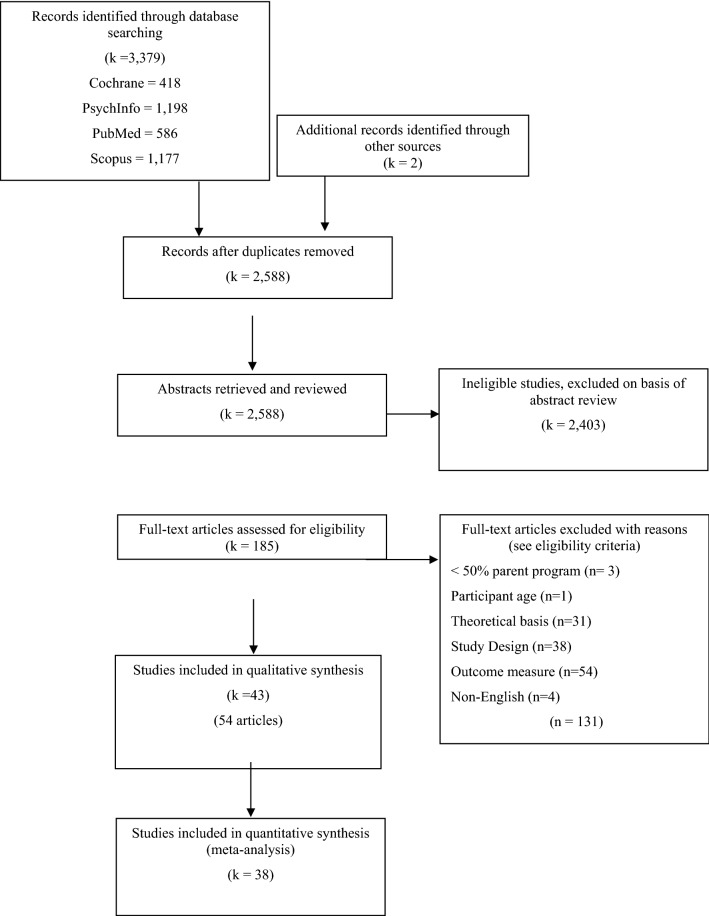
Flowchart of included and excluded studies

Electronic database searches yielded 3379 records (see Fig. 1). Two additional articles were retrieved through hand-searching. After duplicates were removed, 2588 records remained, and following screening of title and abstracts, 185 full-text articles were assessed for eligibility. Inter-rater reliability for the initial inclusion of full-text articles was 97%. Forty-three studies (54 papers) met eligibility criteria for qualitative analysis, with 38 included in quantitative synthesis.^1^ The references of all included papers are provided in Appendix C (Online Resource 1).

Details of study participants, interventions, comparators, and outcome measures in the 43 included studies are presented in Appendix D (Online Resource 1). Three studies were quasi-experimental (Becker-Weidman, 2006; Giannotta et al., 2013; Katz et al., 2020), the remaining were RCTs. Studies included 5542 children aged from 0 to 18 years (*M* = 7.14) and their caregivers. The majority of studies (*K* = 21) included birth parents, though studies also included adoptive (*K* = 6), foster (*K* = 2) and mixed samples of caregivers, including kinship caregivers (*K* = 5). Nine studies did not explicitly report the caregiver relationship to child participants, though they are likely birth parents. Participants were from community (34.88%), clinical (27.91%), maltreatment-exposed (30.23%), and ‘other’ samples (6.98%; e.g., parent with depression). Studies were conducted in USA, Australia, Italy, Germany, Sweden, Norway, Netherlands, and Iran. Sixty-five percent of children’s ethnicity was unknown, with the majority known being White (13%) and African American (10%). Fifty-nine percent of parents’ ethnicity was unknown, with the majority known also White (29%) and African American (6%).

Twenty-one AE were included, with eleven of these in a group format. Length of sessions ranged from 3 to 78 sessions (*M* = 13.74). Facilitators included program developers, psychologists, PhD/Masters level students, allied health professionals (e.g., social workers, occupational therapists, and nurse practitioners), and psychiatrists. It was common for interventions to be delivered by postgraduate students who were supervised by program developers. Some studies provided details on level of training conducted, amount of supervision, and measures used to ensure fidelity. These details were used to provide data to measure intervention fidelity discussed later.

Regarding comparator conditions, 19 studies compared AE against a waitlist control, and 26 studies compared AE against an active control, two of these, BPT. No studies compared AE against BPT on INT.

The majority (74%) of studies included parent-report; however, child self-report, teacher-report, and clinician-reported outcomes were also included. Follow-up ranged from immediately post-intervention to 8 years. Thirty-one studies included EXT and 25 included INT. Most commonly, these measures were broadband measures (e.g., CBCL, Achenbach, 1994), though some studies had more narrowband measures such as the BDI-II (Beck et al., 1996). Three studies (Baker et al., 2015; Opiola et al., 2018; Weihrauch et al., 2014) included a combined EXT/INT measure (e.g., CBCL Total Problems) and two studies (Dozier et al., 2006; Lind et al., 2014) did not have the necessary statistics for meta-analysis and hence were only considered for qualitative review.

### Risk of Bias

**Fig. 2:**
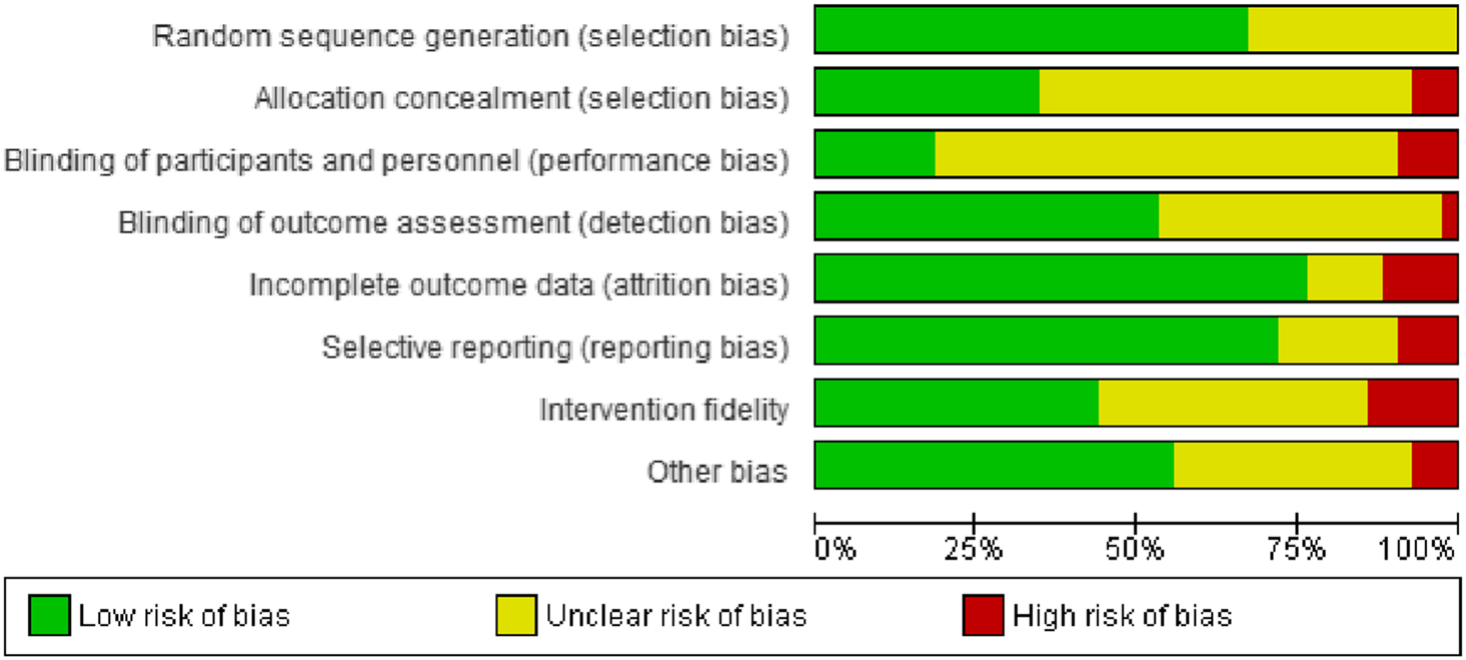
Risk of bias across studies. *Note* Other bias refers to the bias within a study that did not appropriately fit in with one of the other categories. Typically, this referred to studies reporting limited information about participants

Figure 2 shows risk of bias across included studies according to the Cochrane Risk of Bias Tool (Higgens et al., 2011). Assessment of risk of bias indicated that 13 studies were considered to have low risk of bias, 18 were unclear, and 12 (including the three non-randomized studies) were considered high risk of bias performance bias was unclear in the majority of studies (70%), albeit this was not unexpected (Munder & Barth, 2017). Of most interest, six and five studies represented a high risk of bias for intervention fidelity and attrition bias, respectively. Across risk of bias indicators in Fig. 2, intervention fidelity showed the highest risk of bias. This suggests that not all studies may have delivered interventions as intended by program developers or at least assessed and reported whether this was the case. See Appendix E (Online Resource 1) for complete risk of bias details.

### Relative Effects of Attachment and Emotion-Focused Interventions

#### Waitlist Comparators

**Fig. 3:**
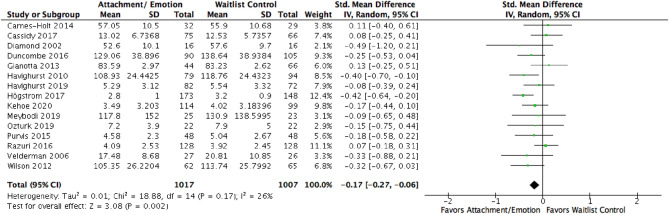
Forest plot of attachment- and emotion-focused parenting interventions versus waitlist controls on externalizing behavior. *Note* A negative *SMD* (left of forest plot) refers to favoring the intervention condition, whereas, a positive *SMD* (right of forest plot) refers to favoring the control condition

**Fig. 4:**
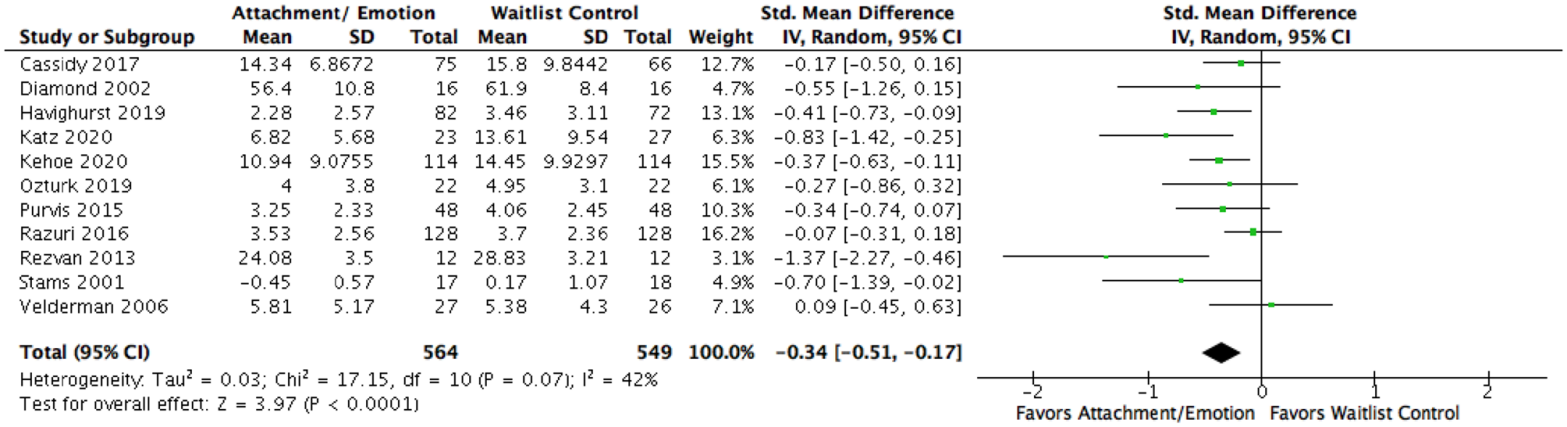
Forest plot of attachment- and emotion-focused parenting interventions versus waitlist controls on internalizing behavior. *Note* A negative *SMD* (left of forest plot) refers to favoring the intervention condition, whereas, a positive *SMD* (right of forest plot) refers to favoring the control condition

**Fig. 5:**
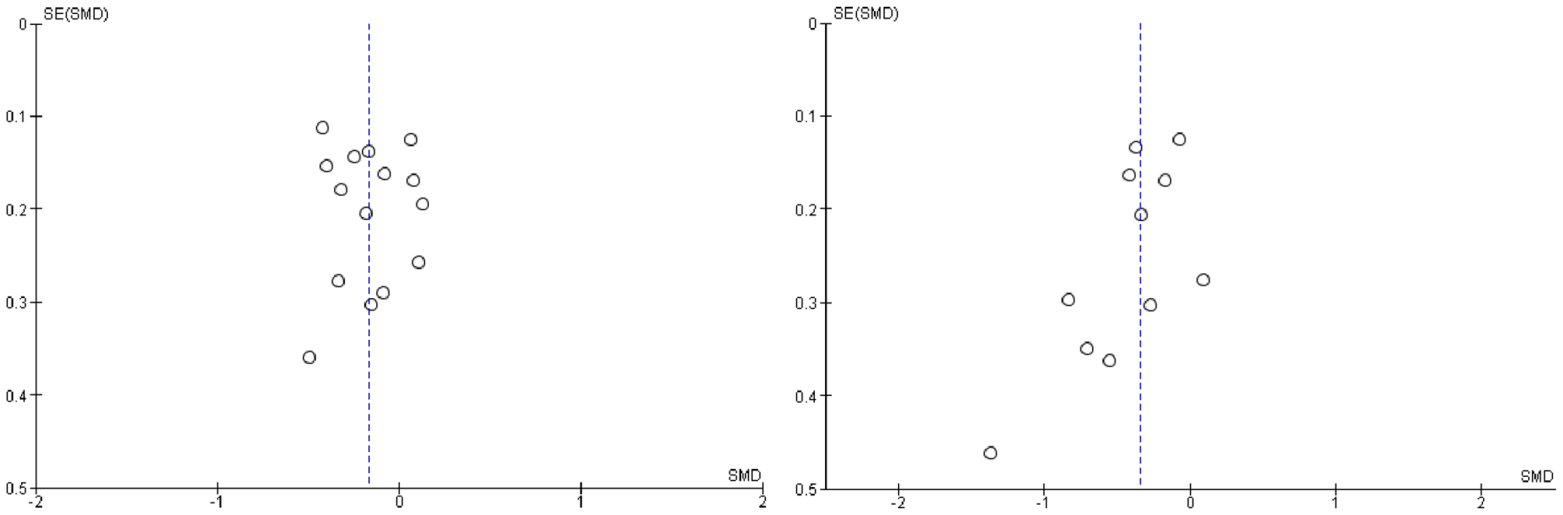
Funnel plots for externalizing and internalizing outcomes when attachment- and emotion-focused parenting interventions are compared to waitlist controls. *Note* Left funnel plot shows studies with externalizing outcomes and the right funnel plot shows studies with internalizing outcomes

To address the first aims, the effects of AE were compared to waitlist controls on EXT and INT (see Figs. 3 and 4 respectively). Fifteen studies were included for EXT, resulting in a pooled *SMD* of − 0.17, 95% CI [− 0.27, − 0.06]. Eleven studies for INT pooled a *SMD* of − 0.34 [− 0.51, − 0.17]. This indicated a small effect in favor of AE on EXT and a small-moderate effect in favor of AE on INT. Visual inspection of the resulting funnel plots was not suggestive of publication bias for EXT. One study (Rezvan et al., 2013) appeared to be an outlier for INT (see Fig. 5), though its upper bound was not lower than the pooled lower bound. A sensitivity analysis was conducted with and without this study. Excluding the study resulted in a *SMD* of − 0.30 [− 0.44, − 0.15], still indicating a small-moderate effect in favor of AE.

#### Behavioral Interventions as Comparators

Next, we aimed to examine the effectiveness of AE for EXT and INT compared to BPT. This aim could not be investigated using quantitative synthesis as there were only two studies that compared AE to BPT on EXT (Duncombe et al., 2016; Högström et al., 2017) and no studies on INT. Thus, we report on the findings of these two papers through qualitative synthesis.

In a sample of 320 4- to 9-year-olds (*M* = 7, *SD* = 1.0) with clinical levels of behavioral difficulties, Duncombe et al. (2016) compared Tuning in to Kids (i.e., an AE; Havighurst et al., 2010) to Triple P-Positive Parenting Program (i.e., BPT; Sanders, 1999). Results demonstrated that both programs showed significantly reduced EXT at 6-month follow-up compared to a waitlist control; however, the two interventions did not significantly differ in their reduction of EXT, *SMD* = 0.10 [− 0.17, 0.38]. Moreover, child age moderated the link between intervention type and EXT. That is, children 8 or older showed greater reductions in EXT at follow-up, than those 7 and younger for the emotion-focused intervention. Conversely, for BPT, children aged 7 and younger showed greater reductions in EXT at follow-up than the older children. The study also found that gender and severity of initial behavior did not moderate the link between the interventions and EXT (Duncombe et al., 2016).

Stattin et al., (2015) compared four parenting interventions in a group of 907 children with EXT aged 3–12 years. Three of these interventions were considered BPT: Comet (Kling et al., 2010), Community Parent Education Program (COPE; Cunningham et al., 1995), and The Incredible Years (IY; Webster-Stratton, 2005); and one, an attachment-based intervention, Connect (Moretti et al., 2018). Comet and COPE included all ages, whereas IY included under 8 only, and Connect included 9- to 12-year-olds. At post-treatment, between-group effects sizes indicated that Connect showed significantly less reductions in EXT to Comet, *SMD* = 0.40 [0.21, 0.59]; though, they did not demonstrate differences in reductions of EXT compared to COPE, *SMD* = 0.00 [− 0.19, 0.19]. We did not estimate between-group differences in this review between Connect and IY due to participant age differences (Stattin et al., 2015). This Cohort was then followed for an additional 2 years, with a final sample of 749 3- to 12-year-olds (Högström et al., 2017). The authors indicated that after 1 year, group differences no longer existed, and at 2-year follow-up children who received the Connect group were the only group to demonstrate additional significant reductions in EXT. Between-group effects reduced to non-significance for Connect compared to Comet at 2-year follow-up, *SMD* = − 0.05 [− 0.21, 0.11], and non-significant effects were sustained compared to COPE, *SMD* = − 0.04 [− 0.2, 0.12]. Hence, at 2-year follow-up, there was no evidence of a difference between the AE and BPT interventions.

#### Active Comparators

**Fig. 6:**
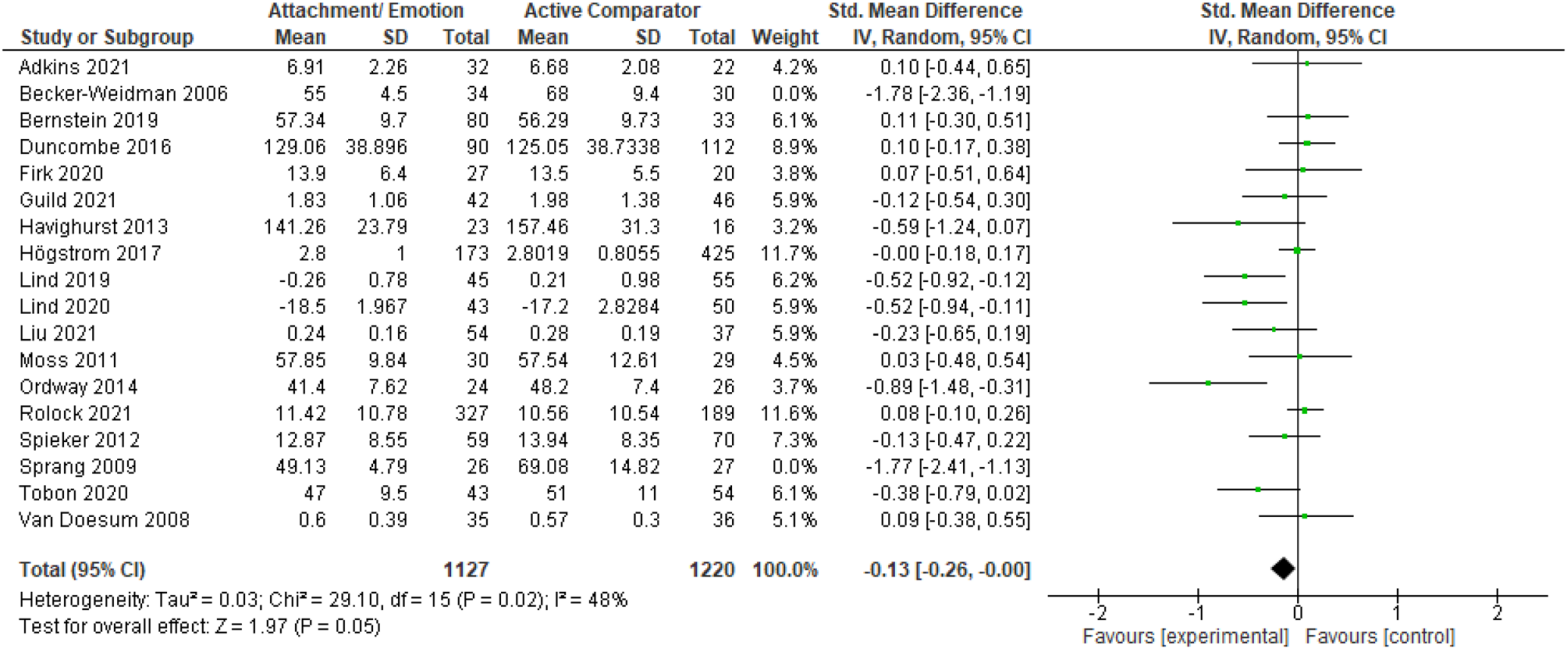
Forest plot of attachment- and emotion-focused parenting interventions versus active comparators on externalizing behavior. *Note* A negative *SMD* (left of forest plot) favors the attachment- and emotion-focused parenting intervention condition, whereas, a positive *SMD* (right of forest plot) favors the active comparator condition. Two outliers were removed (Becker-Weidman, 2006; Sprang, 2009). When these outliers were included, *SMD* = − 0.30, 95% CI [− 0.51, − 0.10], *I*^*2*^ = 80%

**Fig. 7:**
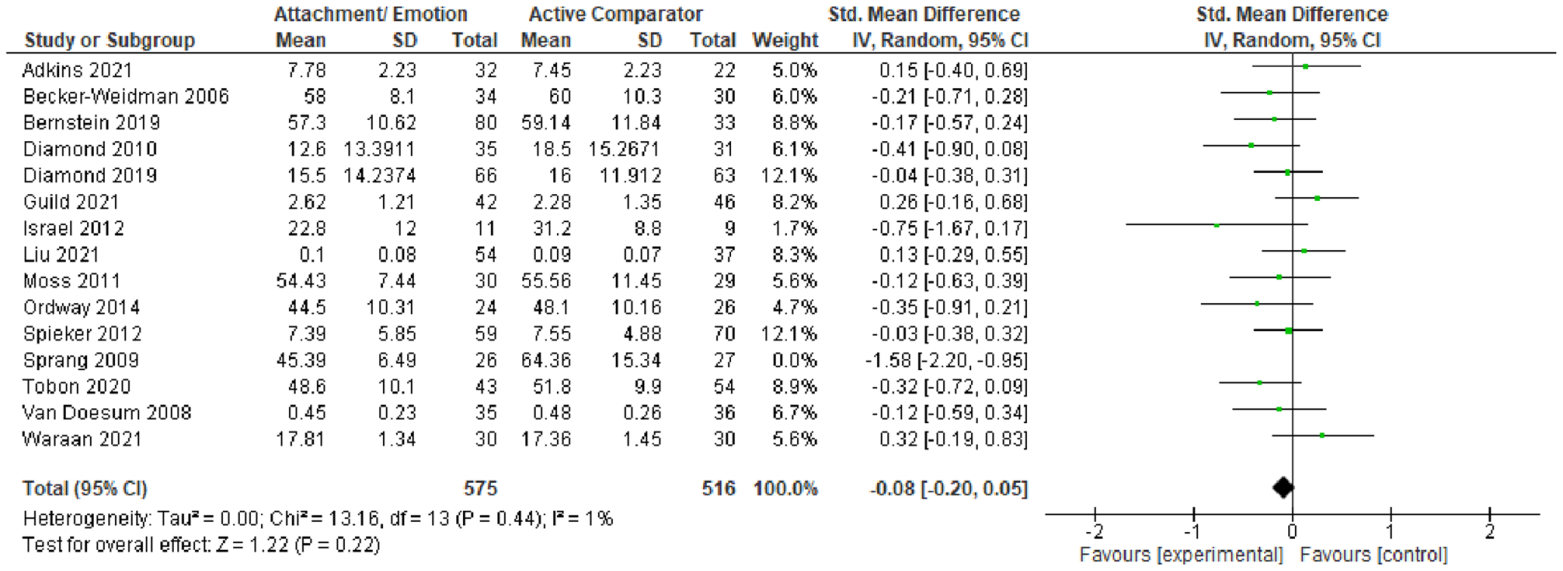
Forest plot of attachment- and emotion-focused parenting interventions versus active comparator on internalizing behavior. *Note* A negative *SMD* (left of forest plot) favors the attachment- and emotion-focused intervention condition, whereas, a positive *SMD* (right of forest plot) favors the active comparator condition. Sprang (2009) was removed from analyses as an outlier. When included, *SMD* = − 0.17, 95% CI [− 0.36, 0.02], *I*^2^ = 60%

**Fig. 8:**
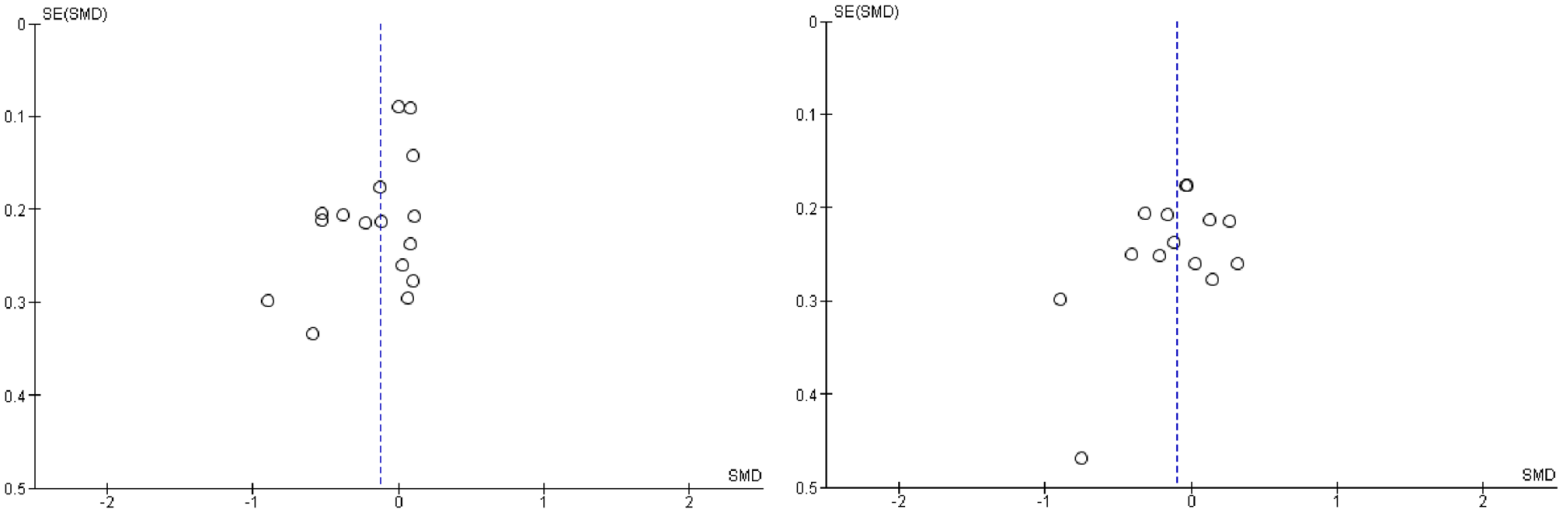
Funnel plots for externalizing and internalizing outcomes when attachment- and emotion-focused parenting interventions are compared to active comparator. *Note* Left funnel plot shows studies with externalizing outcomes and the right funnel plot shows studies with internalizing outcomes

Finally, we aimed to compare AE against any active interventions for EXT and INT (see Figs. 6, 7, and 8). These included TAU, case management, and psychoeducation. Statistical heterogeneity was considered too high to synthesize data quantitatively for EXT (*I*^*2*^ = 80%, and *χ*^2^ = 86.43, *p* < 0.001) and INT (*I*^2^ = 60%, and *χ*^2^ = 34.69, *p* < 0.005). However, when sensitivity analyses were used to remove outliers, heterogeneity reduced for both EXT (*I*^2^ = 48%, *χ*^2^ = 29.10, *p* = 0.02) and INT behaviors (*I*^2^ = 1%, *χ*^2^ = 13.16, *p* = 0.44). The resulting *SMDs* for AE versus active comparators are as follows: *SMD*_EXT_ = − 0.13 [− 0.26, − 0.00, *SMD*_INT_ = − 0.08 [− 0.20, 0.02]. This indicated that against active comparators, there were borderline statistically significant (*p* = 0.05) small effects in favor of AE for EXT, though there was no evidence of a statistically significant effect in favor of AE for INT. Note, the two studies (Becker-Weidman, 2006; Sprang, 2009) removed from these analyses were in favor of AE and when included in synthesis resulted in larger effect sizes for EXT (*SMD* = − 0.30) and INT (*SMD* = − 0.17). These studies were considered clinical populations, non-birth parents and unclear or high risk of bias. Given the clinical heterogeneity among the group of active comparators, these results should be interpreted with caution.

#### Sensitivity Analysis

In addition to examining heterogeneity, sensitivity analysis was used to examine whether removing high of bias risk studies impacted study results. When the first aim was conducted with low risk studies only, the effect size increased for EXT: *SMD*_EXT_ = − 0.30 [− 0.42, − 0.18]*.* However, only two studies were considered low risk of bias for INT, so high-risk studies only were removed to produce a similar result, *SMD*_INT_ = − 0.30 [− 0.46, − 0.15]. Similar results were also found when high-risk studies were removed for AE against active comparators: *SMD*_EXT_ = − 0.09, [− 0.22, 0.03]; *SMD*_INT_ = − 0.11 [− 0.25, 0.04]. For both these comparisons, one study was removed as an outlier that was not considered high risk of bias and was in favor of AE and produced a higher pooled effect size when included, *SMD*_EXT_ = − 0.20, [− 0.38, − 0.01]; *SMD*_INT_ = − 0.26, [− 0.52, 0.01].

#### Subgroup Analyses

We conducted subgroup analyses to consider whether intervention effects changed according to length of follow-up. We also investigated the effects of child age, type of caregiver, baseline clinical severity, delivery method, and sample type; however, there were insufficient studies or heterogeneity was considered too high to consider these factors quantitatively across comparators. Hence, the main analyses were rerun using length of follow-up, while the remainders were considered qualitatively. When age was examined qualitatively, there were mixed findings across studies, such that there was no distinct pattern that emerged to suggest younger or older children showed more favorable treatment outcomes. It is possible that preschool and school-aged children may have benefited more, though there were disproportionate numbers of studies targeting various age groups. The majority of studies included in the review targeted young children (i.e., aged from 3 to 12), with the majority (approximately 82%) of these reporting significant improvements in EXT or INT. Adolescents appeared to have a low proportion of studies that reported significant outcomes in EXT (33%) and INT (14%) following an AE, though there were only seven studies with adolescent participants. Similarly, only one of seven studies targeting infants under the age of 3 reported a significant improvement (in EXT) following an AE. Qualitative review indicated that more studies with non-birth parents (75%) than birth parents (57%) showed significant reductions in EXT or INT; however, there were only 9 out of 43 studies that reported non-birth parents. Nineteen papers reported baseline elevations in EXT or INT, of which the majority reported significant reductions in EXT or INT. Seventy-five percent of papers where children were reported to have clinically elevated EXT, and 60% of papers where children were reported to have clinically elevated INT at baseline, reported a significant change post-treatment. No clear pattern was observed for delivery method, with neither group nor individual delivery of intervention appearing to fare better on treatment outcomes. Sample type suggested that 75% of clinical samples reported a significant improvement in EXT or INT, while 69% of maltreatment-exposed and 60% of community samples reported a significant improvement in EXT or INT. In particular, there appeared to be substantially more clinical samples compared to community samples that reported significant reductions in EXT (83% versus 45%).

Eighteen studies investigated the effects of AE at 6-month follow-up or longer. The main analyses were rerun examining follow-up measures only. When AE were compared to waitlist controls for EXT assessed 6 months or longer after treatment, the effect size was larger: *SMD* = − 0.26 [− 0.38, − 0.13], though this increase was not statistically significantly different from post-treatment effects (*p* = 0.19). For INT, results remained unchanged at 6-month or greater follow-up: *SMD* = − 0.35 [− 0.56, 0.13], *p* = 0.89. In comparison with active comparators, effects at follow-up increased for EXT to statistical significance, *SMD* = − 0.16 [− 0.31, − 0.01], *p* < 0.05; though the test of subgroup difference confirmed that there was no statistically significant change between post-intervention and follow-up effects (*p* = 0.61). There was also no subgroup difference between post-treatment and follow-up for INT compared to active comparators (*p* = 0.39), with the *SMD* remaining not statistically significant at 6-month follow-up, − 0.18 [− 0.45, 0.09].

In addition to planned subgroup analyses, we conducted an exploratory subgroup analysis on AE that specifically targeted child mental health difficulties compared to those that did not [see Appendix F (Online Resource 1)]. For AE that were considered to target mental health, compared to waitlist control, a *SMD* = − 0.23 [− 0.34, − 0.11] was observed for EXT, whereas those that did not target mental health, a *SMD* = − 0.01 [− 0.18, 0.16] was observed. The difference between these subgroups was statistically significant (*p* < 0.01). The same pattern was found for INT, the pooled SMD was statistically significantly (*p* < 0.01) greater for interventions designed to target child mental health outcomes, *SMD* = 0.49 [− 0.71, − 0.28] from those that did not *SMD* = − 0.17 [− 0.35, 0.01]. Heterogeneity was considered too high within subgroups to examine active comparators for EXT, and subgroup analysis for INT demonstrated no statistically significant difference (*p* = 0.56). See Appendix G (Online Resource 1) for complete details of subgroup and sensitivity analyses conducted.

#### Secondary Outcomes

**Fig. 9:**
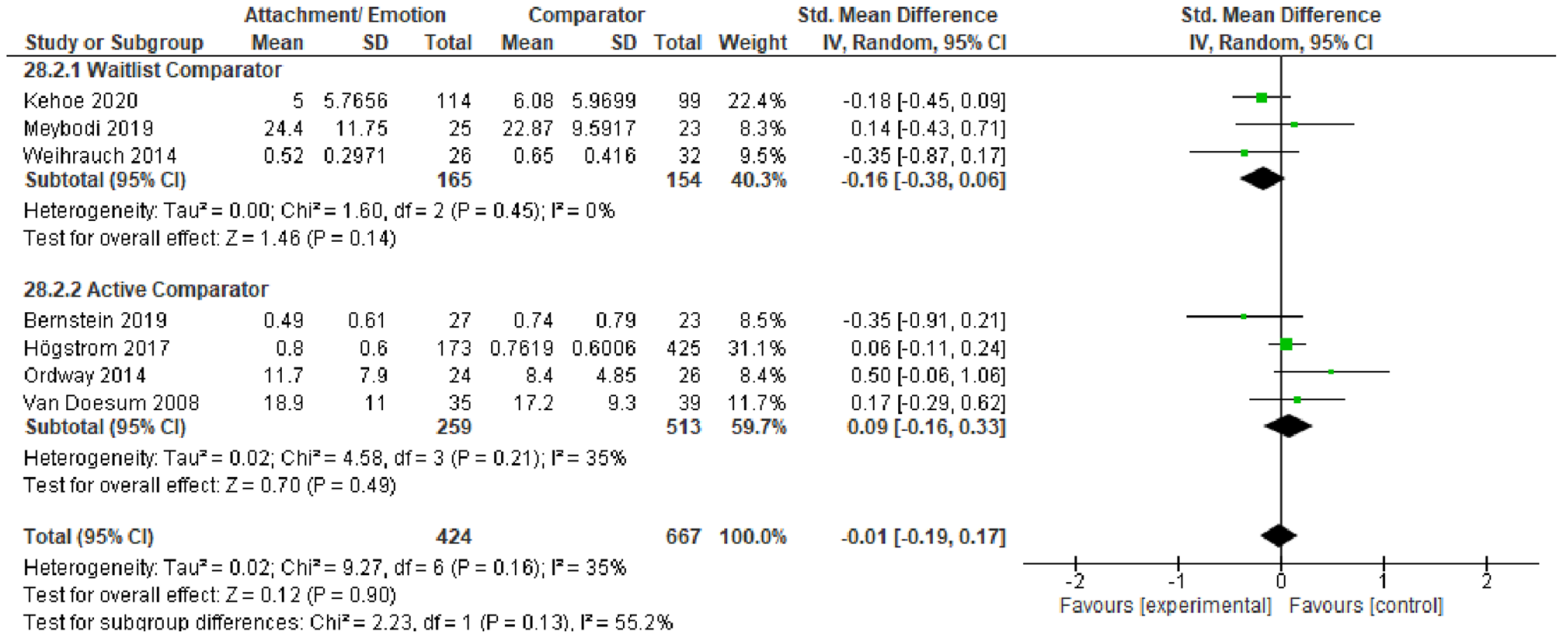
Forest plot of attachment- and emotion-focused parenting interventions versus waitlist and active comparators on parent mental health outcomes. *Note* A negative *SMD* (left of forest plot) favors the attachment- and emotion-focused intervention condition, whereas, a positive *SMD* (right of forest plot) favors the comparator condition

Our final analysis investigated AE effects on secondary parent outcomes. Two main categories were of relevance: parent mental health—i.e., anxiety and/or depressive symptoms—(*k* = 7) and parent stress (*k* = 7) outcomes. Parent mental health outcomes were pooled into a meta-analysis by subgroup of waitlist and active comparators (see Fig. 9). A synthesis of three studies against waitlist controls indicated non-statistically significant effects on parent mental health outcomes, *SMD* = − 0.16 [− 0.38, 0.06]. Similarly, four studies against active comparators also indicated no effect of AE on parent mental health outcomes, *SMD* = 0.09 [− 0.19, 0.33]. Due to limited studies and high heterogeneity, we were unable to pool parenting stress outcomes through meta-analysis. Five of these studies were in favor of AE in reducing parenting stress (Adkins et al., 2021; Baker et al., 2015; Firk et al., 2020; Opiola & Bratton, 2018; Sprang, 2009) and two found no statistically significant difference between AE and the comparator condition in reducing parenting stress (Ozturk et al., 2019; Spieker et al., 2012).

## Discussion

To date, BPT has been by far the most established model of parenting intervention for reducing child mental health problems, particularly EXT, and the predominant focus of treatment outcome research in the area. Notwithstanding this, the popularity of AE appears to have grown among clinicians, researchers, and parents. This systematic review and meta-analysis aimed to conduct the first examination of the effectiveness of AE on EXT and INT compared to (1) waitlist comparators, (2) BPT, and (3) any active comparators. We also conducted additional planned analyses on risk of bias and length of follow-up, as well as analyzed parent mental health as a secondary outcome.

Meta-analytic findings supported our first aim and suggest that AE are more effective at reducing EXT and INT compared to waitlist conditions. Although we were unable to test our second aim using meta-analysis, findings from our narrative review of two RCTs suggest that AE may produce comparable effects to BPT for decreasing EXT. No retrieved studies directly compared AE to BPT on INT; thus, it is unknown how the two interventions compare in this regard. AE did not show to be more effective than active comparators for EXT or INT, not supporting our third aim. However, additional analyses found that at 6-month follow-up or greater, effects for INT were sustained and non-significantly increased for EXT, such that at 6-month follow-up, AE showed a significant small effect relative to active comparators for EXT. Sensitivity analyses also found that when high risk of bias studies were removed, effect sizes for EXT increased relative to waitlist comparators and remained consistent for INT across comparators. Interestingly, no statistically significant effect was observed between AE and any comparator for parent mental health outcomes.

The current findings both concur with and diverge from results from previous meta-analyses investigating intervention effects on EXT and INT. A meta-meta-analysis of various parenting interventions for clinic-referred children 13 and under found an effect size of 0.46 for EXT (Mingebach et al., 2018), which is higher than the effect size observed in our study. This difference in effect size could be a true estimate, though it could also relate to several other factors. First, our review paper spans across birth to 18, whereas this prior meta-analysis only looked at preadolescence, missing an additional 6 years where problem behaviors can increase (Moretti et al., 2018). Second, considerable heterogeneity (*I*^2^ = 84.56%) was observed in the Mingebach et al. (2018) study, whereas we did not conduct a meta-analysis if heterogeneity was considered high (*I*^2^ > 59%) (Higgens et al., 2021). Third, we included both community and clinical samples, rather than only clinical samples, and past research demonstrates larger effect sizes in clinical samples as higher baseline levels are subject to greater room for change post-treatment (Leijten et al., 2013; McMahon et al., 2021). Fourth, although AE derive from similar theoretical orientation, they are more heterogeneous than models of BPT, which may have led to more varied effects on EXT and INT, reducing the overall effect size. As BPT research on mental health outcomes is far more extensive than AE research, we did not have the same degree of flexibility to consider various subgroup and sensitivity analyses to tease apart differences. Therefore, caution needs to be applied when comparing effect sizes to previous review papers; the only way to truly compare these interventions is for participants to be randomly allocated to BPT or AE within the same study.

Conversely, previous meta-analytic research on parenting interventions for INT, which analyzed studies of children from birth to 18 and excluded effect sizes with high levels of heterogeneity, found an overall effect size of 0.12 for INT (Yap et al., 2016). This effect size is smaller than the one observed in our study and appears comparable in terms of population and statistical method. Hence, this suggests that AE could be a more promising choice of parenting intervention for reducing INT in children and adolescents. Nevertheless, without directly comparing AE to BPT—or other parenting interventions—through a meta-analysis of high-quality RCTs, we cannot assume AE is less effective for EXT or more effective for INT.

Considering our findings within the context of past reviews on BPT, both AE and BPT may effectively reduce EXT and INT; however, there may be unique mechanisms underpinning these different intervention approaches. It is suggested that proximal targets of AE, such as attachment security, reflective functioning, caregiver sensitivity, and parent emotional awareness, may be mechanisms accounting for improvements in EXT and INT (Carlone & Milan, 2020; Havighurst et al., 2020; Kobak et al., 2015; Kok et al., 2013; Wang et al., 2013). For example, individual studies included in the review found that parent sensitivity post-intervention mediated the effect of AE on child EXT (Lind et al., 2020); and improvements in parent emotion socialization mediated youth INT following an AE (Kehoe et al., 2020). Further research should shed light on these and other mechanisms accounting for the effects of AE on EXT and INT, as has been done for BPT with regard to behavioral parenting practices (Forehand et al., 2014). If AE and BPT continue to produce comparable results in future RCTs akin to the two observed in this paper, researchers and clinicians may also consider the extent to which AE and BPT are compatible and complementary. For example, it is theorized that individual differences in attachment patterns may be in part learnt through behavioral principles of classical and operant conditioning (Bosmans et al., 2020), and BPT has been shown to improve attachment-based parenting domains such as caregiver sensitivity (O’Connor et al., 2013). Although there have been recent attempts by researchers to integrate these theoretical models in parenting interventions, they have not always found superior effects on EXT (Leijten et al., 2018; O’Hara et al., 2019) and are yet to be examined for INT. Thus, the current review highlights several research directions to examine both possible underlying mechanisms of AE as well as their compatibility with other parenting interventions.

Our review explored several possible factors that may influence or moderate the effectiveness of AE on EXT and INT. Firstly, follow-up analyses demonstrated a non-statistically significant increase in EXT from post-treatment to 6-month follow-up or greater, and effects at post-treatment held at follow-up for INT. This suggests that at minimum, meta-analytic evidence demonstrates that the effects of AE remain beyond immediate post-intervention for both EXT and INT. Previous research suggests that AE may produce sleeper effects (Bakermans-Kranenburg et al., 2003). For example, a study in our review that compared an AE (Connect; Moretti et al., 2018) to several BPT interventions found that while BPT showed the greatest effect post-treatment, the AE continued to show improvements at 2-year follow-up such that there were no longer differences in effect sizes between BPT and AE (Högström et al., 2017). If future research confirms sleeper effects, this could be unique to AE relative to the broader parenting intervention literature. For example, a review of 40 RCTs found that BPT had sustained effects—effects sizes remained the same—for up to 3 years without further improvements (van Aar et al., 2017). Regardless, at present, our meta-analysis of 38 studies supports comparable sustained effects at follow-up for AE.

Secondly, study risk of bias appeared to impact AE outcomes, such that low-risk studies produced results that are more favorable for AE compared to waitlist comparators on EXT and INT. Only 13 studies within this review were considered low risk of bias indicating an important limitation in the existing literature, which may also understate the overall effects of AE on EXT and INT.

The current review also attempted planned subgroup analyses including sample type, age, caregiver type, delivery method, and baseline levels of EXT and INT; however, heterogeneity and/or limited studies prevented consistently quantitatively synthesizing results. Individual study findings suggested trends toward clinical samples and higher baseline levels of EXT/INT showing greater change following AE relative to comparators. These trends are comparative to previous research that has found that parenting interventions are more effective in decreasing problem severity when initial problem severity is higher (Leijten et al., 2013; McMahon et al., 2021). A greater proportion of studies with non-birth parents relative to birth parents showed more favorable results, though there were very limited studies to determine a pattern. Previous research has been inconclusive as to whether socioeconomic characteristics are potential moderators of intervention effectiveness (McMahon et al., 2021). Regarding our qualitative results, it appeared that AE may have showed more favorable treatment outcomes for preschool and school-aged children relative to infants and adolescents. However, we are cautious in interpreting this potential pattern of results as there were limited studies with children aged under 3 or over 12 years. Thus, this is an important area for further research. It is also important to note that previous research has been mixed on whether age is a moderator on psychosocial interventions for children and adolescents (e.g., McMahon et al., 2021). Finally, no clear patterns emerged for the impact of delivery method on treatment outcomes.

In addition to planned analyses, exploratory subgroup analyses found that AE specifically developed to target child and adolescent mental health outcomes showed more favorable outcomes for EXT and INT. These AE tended to include components or modules that focused on parent–child conflict and/or parenting skills to manage difficult child behavior. Future research should investigate which specific strategies included in AE may fuel greater reductions in EXT or INT, in addition to understanding what mechanisms may explain these effects. Microtrials of common components in AE to isolate mechanisms of effects could be utilized to this end (e.g., see Leijten et al., 2015).

In addition to child EXT and INT, our review investigated secondary parent mental health and wellbeing outcomes, which included depression, anxiety, and stress. Parent mental health outcomes—most typically, depression—were quantitatively synthesized, though surprisingly, no statistically significant effects were observed for AE against any comparators. This is inconsistent with a previous meta-analysis of an AE that found reductions in parent depressive symptoms (Yaholkoski et al., 2016); however, other AE studies and parenting program research have also found null findings (Baradon et al., 2018; Jeong et al., 2021). Previous research suggests that parent training programs may significantly improve parent variables that are most proximal to the intervention such as parenting stress and perceived parenting competence rather than distal outcomes such as depression (Colalillo & Johnston, 2016). Hence, it could be hypothesized that if interventions more closely targeted parent mental health and wellbeing, greater changes may be observed. Connect is one example of an AE that considered mental health outcomes in its development and has shown positive improvements in this domain (Osman et al., 2017).

Although there were insufficient studies to pool parent stress quantitatively, five of seven studies were in favor of AE reducing parental stress relative to comparators, suggesting potential benefits for improving parental stress. This also highlights an important gap in AE research, in that similar to child outcomes, parent measures are usually focused on attachment-related constructs such as caregiver sensitivity (Steele & Steele, 2018), and effects on parental mental health are scarcer.

## Limitations

To the best of our knowledge, this is the first quantitative synthesis of the effectiveness of AE for child and adolescent EXT and INT. Our review overcomes shortcomings of previous studies by taking a comprehensive and inclusive approach to ensure all controlled trials of published AE that assessed EXT and INT post-treatment were included. We were intentionally broad in our included presentations, caregivers, age range, and other demographic variables.

Notwithstanding these strengths, this study has several important limitations. First, many studies we included did not require participants to have baseline clinical levels of EXT or INT. Previous research has demonstrated that baseline EXT moderates treatment effects, such that greater reductions in EXT are linked to higher baseline EXT (Leijten et al., 2013; McMahon et al., 2021). Due to insufficient studies, missing baseline data, and heterogeneity, our review was unable to quantitatively investigate whether baseline clinical level was a moderator across comparators. Second, five studies meeting inclusion criteria for this review were unable to be used in quantitative analysis due to missing data that were not provided by authors when requested. Third, 12 included studies were considered to have a high risk of bias, which is a limitation of intervention research. In our study, removing studies with high risk of bias, including non-randomized studies, did not appear to reduce overall effect sizes; however, this is still a potential issue that should be considered in future research. In particular, intervention fidelity was rated the highest risk of bias meaning that we were unsure of the extent to which some studies may have delivered the intervention as program developers intended it. Fourth, the current review chose a common recommended statistical approach for meta-analysis that relies on the assumption that the control and intervention groups have similar baseline characteristics (Cuijpers et al., 2017). Given the risk of bias found, this may not have consistently been the case and could have affected the pooled effect sizes. Since lower risk of bias studies showed more favorable outcomes, it is possible that treatment effects in this review are underestimated. Fifth, while this meta-analysis did not report results if statistical heterogeneity was high, there was clinical heterogeneity among types of active comparators. We followed planned protocol by pooling all active comparators, though there were insufficient studies to comprehensively investigate subgroups by type of active comparator. Further research needs to consider relative efficacy of AE to other treatments, including BPT as more RCTs are published. Finally, this meta-analysis primarily relied on parent-report outcomes (75% of included studies), though child-report, teacher-report, or observational data may have shown different results (e.g., see Fearon et al., 2010).

## Research and Clinical Implications

Findings from this review offer new and important insights to inform both research and clinical practice in working with families. Prior results support AE in improving caregiver sensitivity, reflective functioning, and child attachment security, with a particular emphasis on children who have been exposed to maltreatment (Bakermans-Kranenburg et al., 2003; Dozier et al., 2011). Building on this evidence base, our results provide strong support for AE as an appropriate intervention approach for effectively reducing EXT and INT in children and adolescents. While there is still limited understanding regarding the relative efficacy of AE to other intervention programs, the current findings may help inform evidence-based clinical decision making regarding the type of parenting intervention that may best suit families’ needs and preferences (Lilienfeld et al., 2013; Spring, 2007).

Future research would benefit from additional comparative trials, directly examining the relative effects of AE against BPT on child EXT and INT, as well as possible child and family characteristics that may moderate AE effects on these outcomes. Moreover, where AE research is undertaken, mental health outcome measures should be regularly incorporated, and specifically, independent measures of EXT and INT are recommended (see Achenbach et al., 2016). In addition to analyzing effectiveness, we also strongly recommend investigating practitioner and client acceptability of AE. While this meta-analysis demonstrates statistically significant reductions in EXT and INT following AE, it is unknown what mechanisms may explain this. Future research should investigate this topic in addition to exploring the extent to which AE and BPT interventions may be compatible or superior as an integrated approach versus alone.

## Conclusion

In summary, this study provides initial meta-analytic evidence for the effectiveness of AE for reducing child and adolescent EXT and INT. Significant small-to-moderate effects were found overall for AE that held for 6-month follow-up and onwards. The current findings provide a significant and timely overview and update for research and practice in parenting interventions. Evidence regarding the relative efficacy of AE to BPT, however, remains limited in the absence of sufficient head-to-head RCTs. Future research is needed to directly investigate the relative effectiveness and acceptability of these two interventions as well as consider the inclusion of EXT and INT more regularly in AE research.

## Supplementary Information

Below is the link to the electronic supplementary material.

Supplementary file1 (DOCX 91 KB)

## Data Availability

The authors declare that all data supporting the findings of this study are available within the article.

## Footnotes

1 Twelve authors were contacted to retrieve the additional data needed to be included in quantitative synthesis; however, only one of these authors was able to provide the necessary data.
