# Correction to: Attachment- and Emotion-Focused Parenting Interventions for Child and Adolescent Externalizing and Internalizing Behaviors: A Meta-Analysis

**DOI:** 10.1007/s10567-022-00403-6

**Published:** 2022-06-30

**Authors:** Samantha Jugovac, Richard O’Kearney, David J. Hawes, Dave S. Pasalich

**Affiliations:** 1 Research School of Psychology, Australian National University, Canberra, Australia; 2 School of Psychology, The University of Sydney, Sydney, Australia

## Correction to: Clin Child Fam Psychol Rev (2022) 10.1007/s10567-022-00401-8

After publication of the original article*,* it came to the authors’ attention that there were errors in the caption of Figs. 2, 3, 4, 5, 6, 7, 8, 9 (i.e., 6 figures in total). The corrected captions can be found in the figures below.

**Fig. 2:**
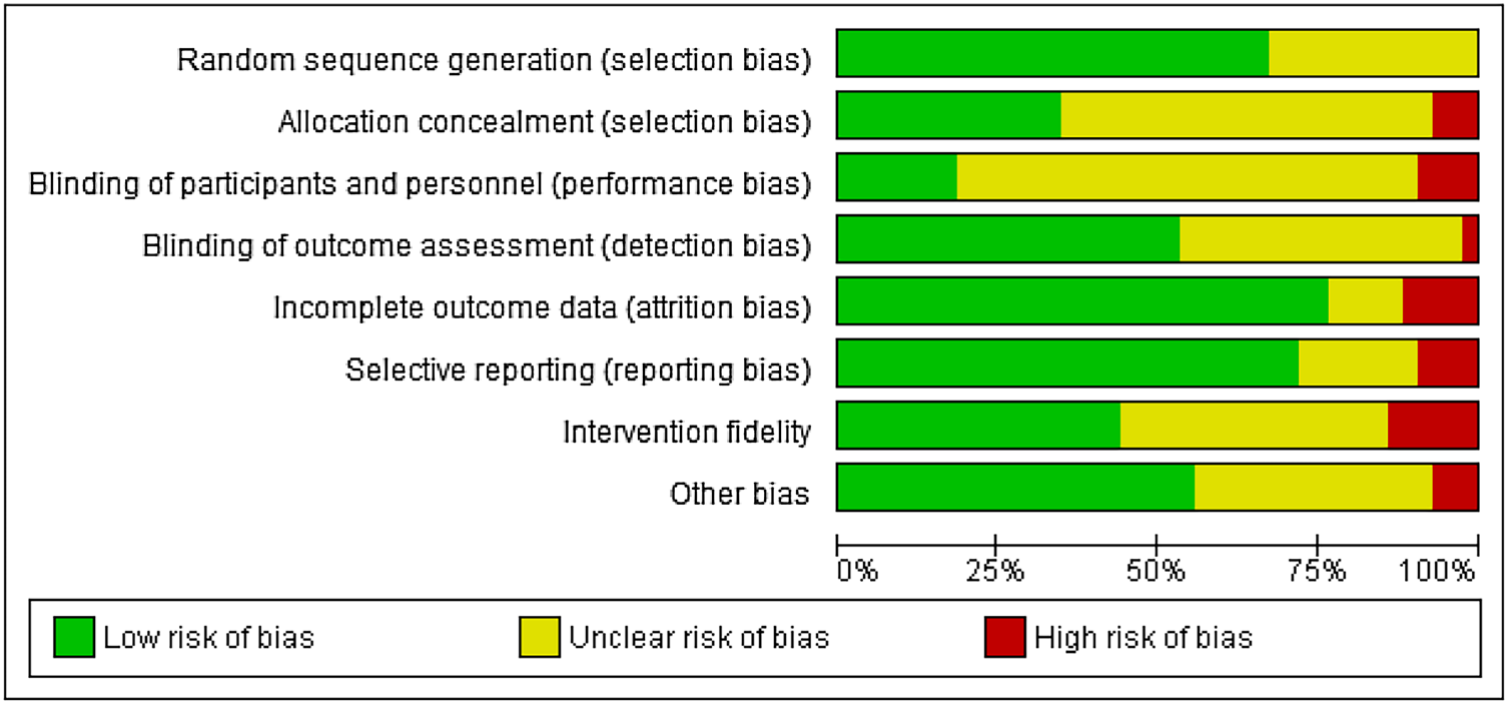
Risk of bias across studies. *Note.* Other bias refers to the bias within a study that did not appropriately fit in with one of the other categories. Typically, this referred to studies reporting limited information about participants

**Fig. 3:**
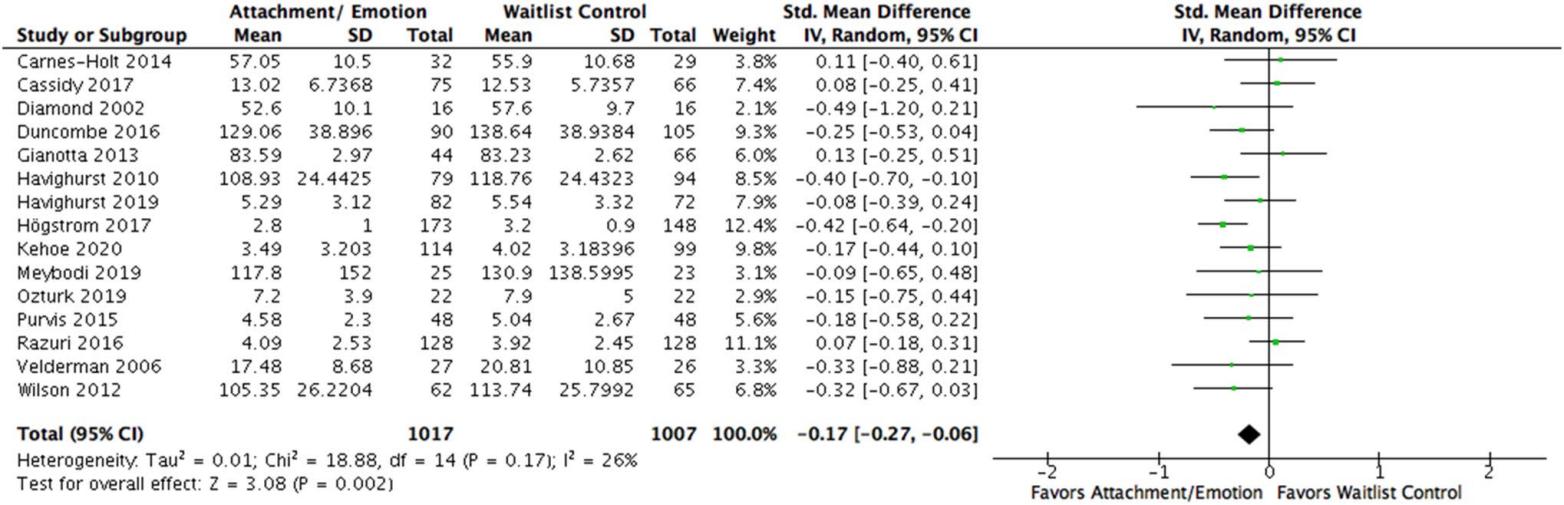
Forest plot of attachment- and emotion-focused parenting interventions versus waitlist controls on externalizing behavior. *Note.* A negative *SMD* (left of forest plot) refers to favoring the intervention condition, whereas a positive *SMD* (right of forest plot) refers to favoring the control condition

**Fig. 4:**
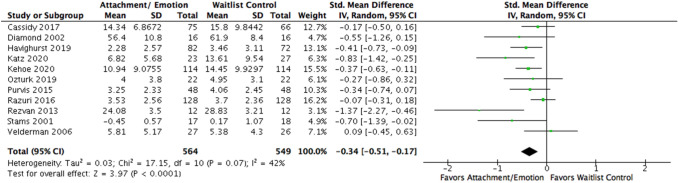
Forest plot of attachment- and emotion-focused parenting interventions versus waitlist controls on internalizing behavior. *Note.* A negative *SMD* (left of forest plot) refers to favoring the intervention condition, whereas a positive *SMD* (right of forest plot) refers to favoring the control condition

**Fig. 5:**
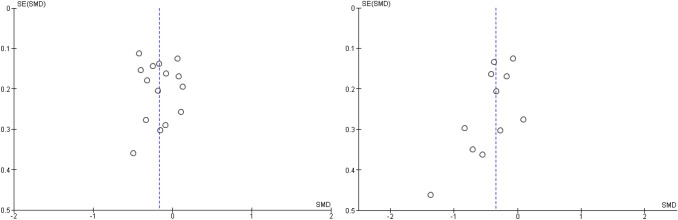
Funnel plots for externalizing and internalizing outcomes when attachment- and emotion-focused parenting interventions are compared to waitlist controls. *Note.* Left funnel plot shows studies with externalizing outcomes and the right funnel plot shows studies with internalizing outcomes

**Fig. 6:**
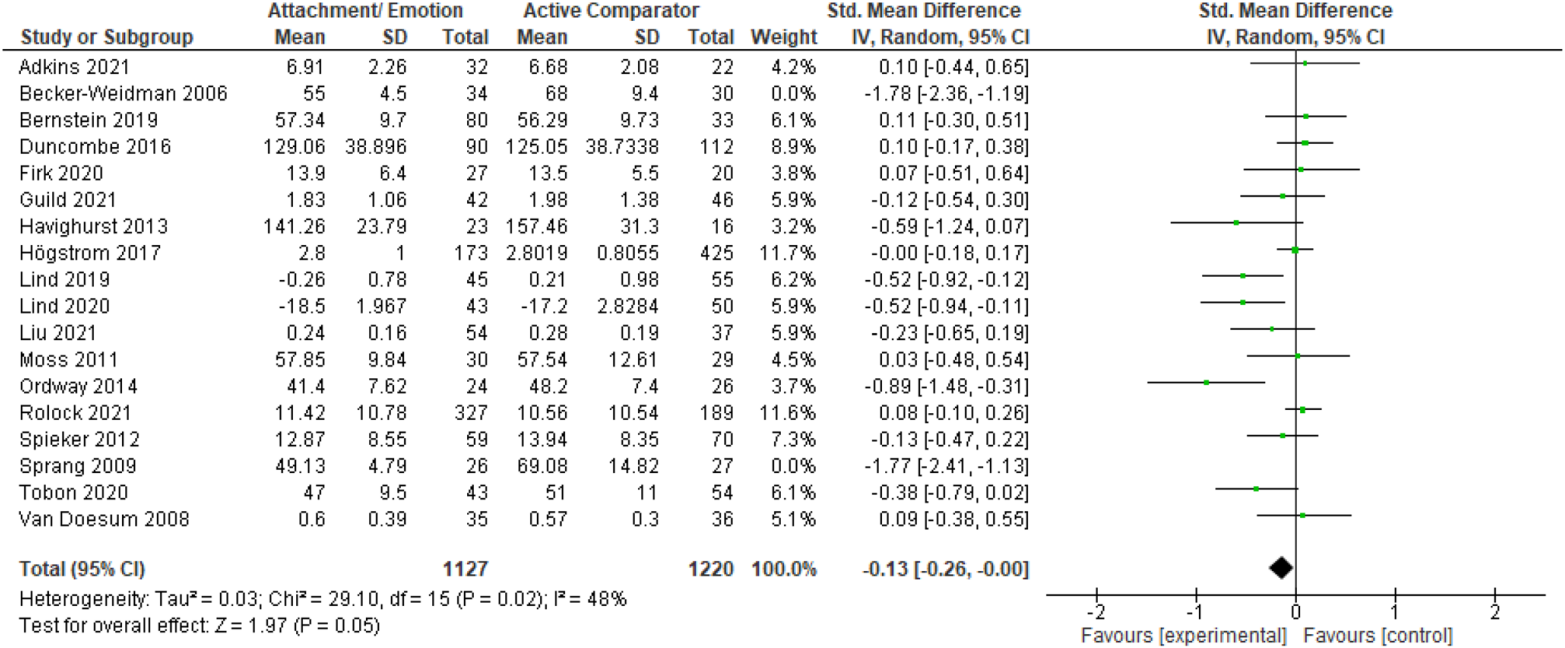
Forest plot of attachment- and emotion-focused parenting interventions versus active comparators on externalizing behavior. *Note.* A negative *SMD* (left of forest plot) favors the attachment- and emotion-focused parenting intervention condition, whereas a positive *SMD* (right of forest plot) favors the active comparator condition. Two outliers were removed (Becker-Weidman et al., 2006; Sprang, 2009). When these outliers were included, *SMD* = − 0.30, 95% CI [ − 0.51,  − 0.10], *I*^*2*^ =  80%

**Fig. 7:**
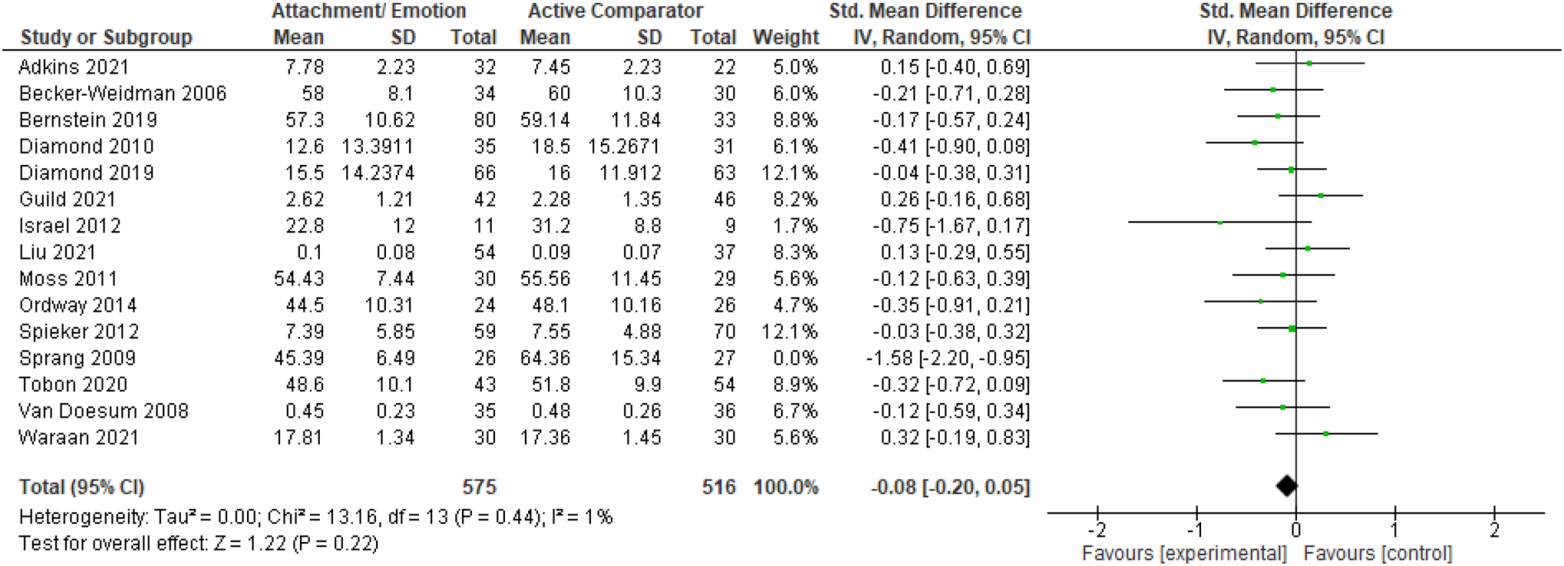
Forest plot of attachment- and emotion-focused parenting interventions versus active comparator on internalizing behavior. *Note.* A negative *SMD* (left of forest plot) favors the attachment- and emotion-focused intervention condition, whereas, a positive *SMD* (right of forest plot) favors the active comparator condition**.** Sprang (2009) was removed from analyses as an outlier. When included, *SMD* = -0.17, 95%CI [-0.36, 0.02], *I*^2^ = 60%

**Fig. 8:**
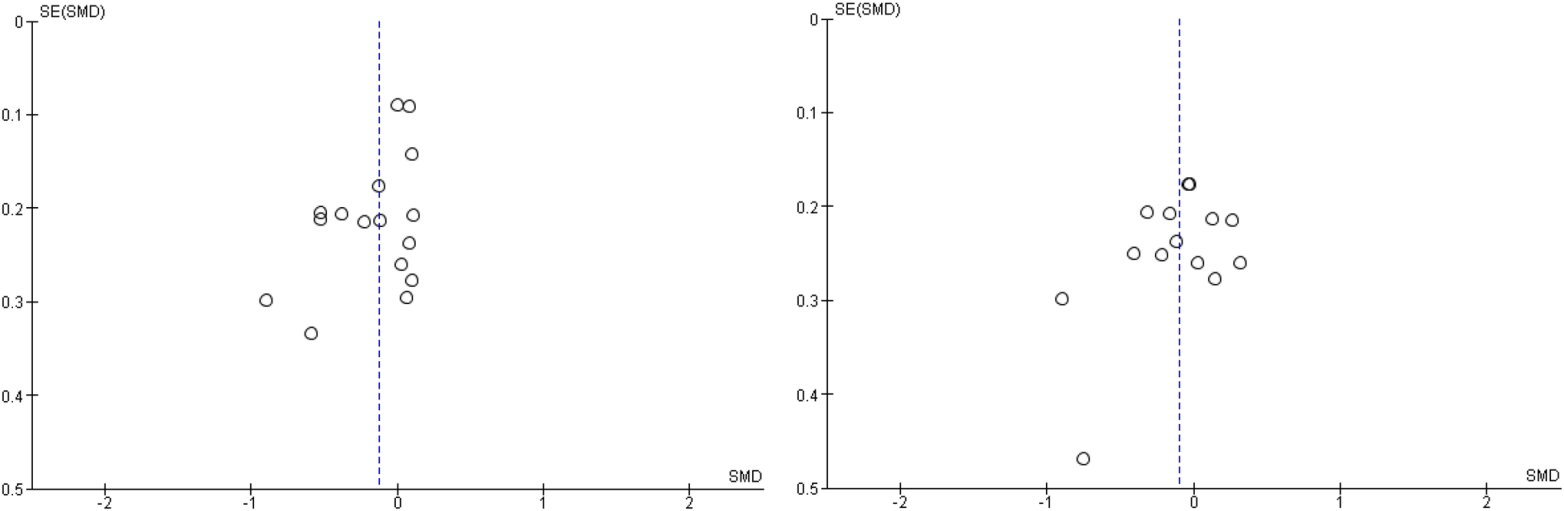
Funnel plots for externalizing and internalizing outcomes when attachment- and emotion-focused parenting interventions are compared to active comparator. *Note.* Left funnel plot shows studies with externalizing outcomes and the right funnel plot shows studies with internalizing outcomes

**Fig. 9:**
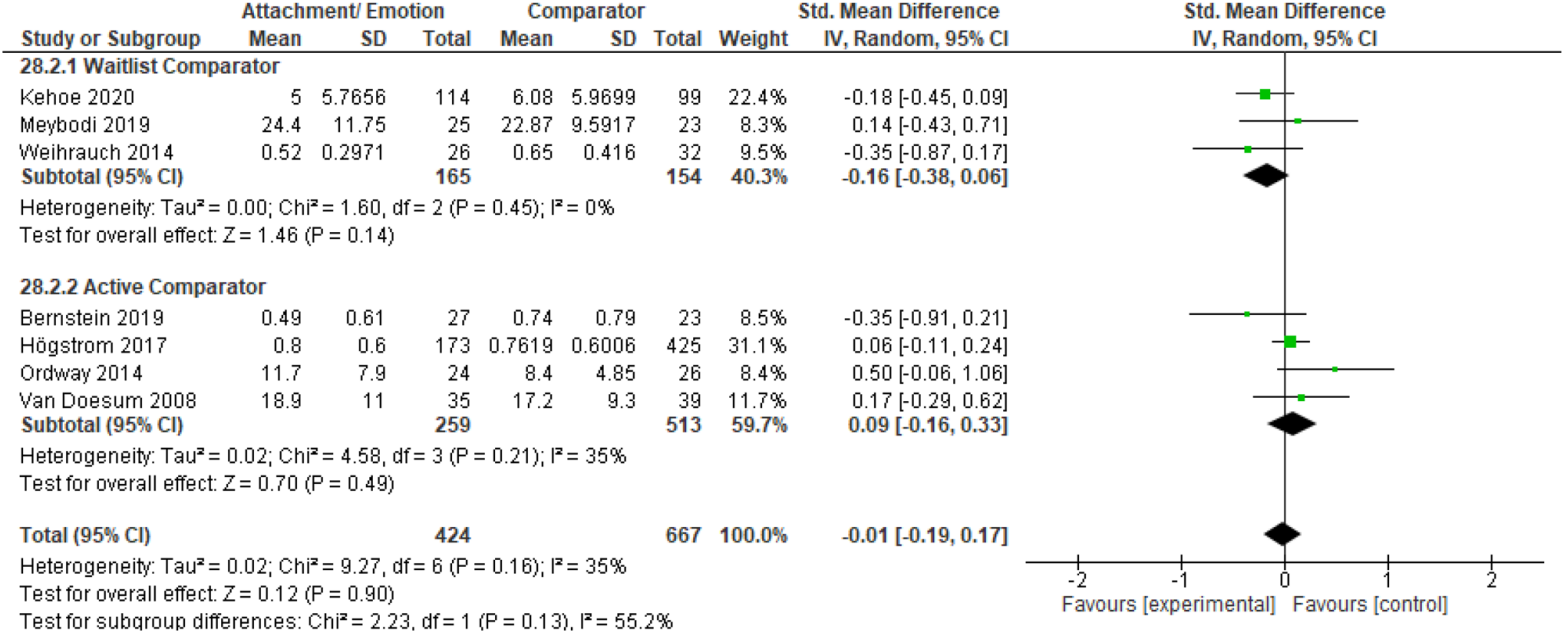
Forest plot of attachment- and emotion-focused parenting interventions versus waitlist and active comparators on parent mental health outcomes *Note.* A negative *SMD* (left of forest plot) favors the attachment- and emotion-focused intervention condition, whereas, a positive *SMD* (right of forest plot) favors the comparator condition

The original article has been corrected.

