# Supplementary material for: Attachment- and Emotion-Focused Parenting Interventions for Child and Adolescent Externalizing and Internalizing Behaviors: A Meta-Analysis
Source: Clin Child Fam Psychol Rev. 2022 Jun 10;25(4):754–73. doi: 10.1007/s10567-022-00401-8 (PMC9622525; doi:10.1007/s10567-022-00401-8)
Supplement: Supplementary file 1 — Supplementary file1 (DOCX 91 KB) [file 10567_2022_401_MOESM1_ESM.docx]

Online Resource 1 for

**Attachment- and Emotion-Focused Parenting Interventions for Child and Adolescent Externalizing and Internalizing Behaviors: A Meta-Analysis**

Clinical Child and Family Psychology Review

Samantha Jugovac, ^1^ Richard O’Kearney, ^1^ David J. Hawes, ^2^ and Dave S. Pasalich^1^

^1^Research School of Psychology, Australian National University, Canberra Australia

^2^School of Psychology, The University of Sydney, Sydney, Australia

Correspondence concerning this article should be addressed to Samantha Jugovac, Research School of Psychology, Building 39, Science Rd Canberra ACT 2601, Australia. Email: [samantha.jugovac@anu.edu.au](mailto:samantha.jugovac@anu.edu.au)

**Appendix A**

Parenting Interventions Categorized as Attachment- and Emotion- Focused, Behavioral or Blended

| Attachment/Emotion | Behavioral | Blended |
| --- | --- | --- |
| Circle of Security (Hoffman et al., 2006)  Tuning in to Kids (Havighurst et al., 2010)  Tuning in to Teens (Havighurst et al., 2015)  Connect (Moretti et al., 2018)  Attachment and Biobehavioral Catch-up (Dozier et al., 2011)  Minding the Baby (Slade et al., 2018) | Helping the Noncompliant Child (Forehand & McMahon, 1981)  Parent-Child Interaction Therapy (Herschell et al., 2002)  Parent Management Training- Oregon Model (Forgatch & Patterson, 2010)  Triple P- Positive Parenting Program (Sanders, 1999)  Hitkashrut’ (Somech & Elizur, 2012)  Strong Families Smart Website intervention – Internet assisted parent-training ([Sourander](https://www.ncbi.nlm.nih.gov/pubmed/?term=Sourander%20A%5BAuthor%5D&cauthor=true&cauthor_uid=26913614) et al., 2016) | The Parenting your Hyperactive Preschooler Program (Herbet et al., 2013)  1-2-3 Magic with emotion coaching (Porzig-Drummond et al., 2014)  Parent Management Training + emotional reminiscing (Salmon et al., 2009)  Video-Intervention Feedback for Promoting Positive Parenting and Sensitive Discipline (Juffer et al., 2018). |

*Note.* This table is not exhaustive and only provides some examples of each of the above intervention categories.

**References**

Dozier, M., Bick, J., & Bernard, K. (2011). Intervening with foster parents to enhance biobehavioral outcomes among infants and toddlers. *Zero to Three, 31*(3), 17-22. <https://www.ncbi.nlm.nih.gov/pmc/articles/PMC3517191/>

Forehand, R. L., & McMahon, R. J., (1981). *Helping the Noncompliant Child: A clinician‘s guide to parent training.* The Guilford Press.

Forgatch, M. S., & Patterson, G. R. (2010). Parent Management Training—Oregon Model: An intervention for antisocial behavior in children and adolescents. In J. R. Weisz & A. E. Kazdin (Eds*.), Evidence-based psychotherapies for children and adolescents* (pp. 159–177). The Guilford Press.

Havighurst, S. S., Kehoe, C. E., & Harley, A. E. (2015). Tuning in to Teens: Improving parental responses to anger and reducing youth externalizing behavior problems. *Journal of Adolescence, 42,* 148-158. <https://doi.org/10.1016/j.adolescence.2015.04.005>

Havighurst, S. S., Wilson, K. R., Harley, A. E., Prior, M. R., & Kehoe, C. (2010). Tuning in to Kids: Improving emotion socialization practices in parents of preschool children - Findings from a community trial. *The Journal of Child Psychology and Psychiatry, 51*(12), 1342-1350. <https://doi.org/10.1111/j.1469-7610.2010.02303.x>

Herbet, S., Harvey, E. A., Roberts, J. L., Wichowski, K., Lugo-Candelas, C. I. (2013). A randomized controlled trial of parent training and emotion socialization program for families of hyperactive preschool-aged children. *Behavior Therapy, 44*(2), 302-316. <https://doi.org/10.1016/j.beth.2012.10.004>

Herschell, A. D., Calzada, E. J., Eyberg, S. M., McNeil, C. B. (2002). Parent-child interaction therapy: New directions in research. *Cognitive and Behavioral Practice, 9*(1), 9-16. <https://doi.org/10.1016/S1077-7229(02)80034-7>

Hoffman, K., Marvin, R. S., Cooper, G. & Powell, B. (2006). Changing toddlers' and preschoolers' attachment classifications: The Circle of Security intervention. *Journal of Consulting and Clinical Psychology, 74*(6), 1017-1026. <https://doi.org/10.1037/0022-006X.74.6.1017>

Juffer, F., Bakermans‑Kranenburg, M. J., & van IJzendoorn, M. H. (2018). Video‑feedback Intervention to Promote Positive Parenting and Sensitive Discipline: Development and meta‑analytic evidence for its effectiveness. In H. Steele & M. Steele (Eds.), Handbook of attachment-based interventions (pp. 1–26). The Guilford Press.

Moretti, M. M., Pasalich, D. S., O’Donnell, K. A. (2018). Connect: An attachment-based program for parents of teens. In H. Steele & M. Steele (Eds). *Handbook of attachment-based interventions* (pp. 375-400). The Guilford Press.

Porzig-Drummond, R., Stevenson, R. J., & Stevenson, C. (2014). The 1-2-3 Magic parenting program and its effect on child problem behaviors and dysfunctional parenting: A randomized controlled trial. *Behaviour Research and Therapy, 58,* 52-64. <http://dx.doi.org/10.1016/j.brat.2014.05.004>

Salmon, K., Dadds, M. R., Allen, J., & Hawes, D. J. (2009). Can emotional language skills be taught during parent training for conduct problem children? *Child Psychiatry and Human Development, 40,* 485-498. <https://doi.org/10.1007/s10578-009-0139-8>

Sanders, M. R. (1999). Triple P-Positive Parenting Program: Towards an empirically validated multilevel parenting and family support strategy for the prevention of behavior and emotional problems in children. *Clinical Child and Family Psychology Review, 2*(2), 71–90. <https://doi.org/10.1023/A:1021843613840>

Slade, A., Simpson, T. E., Webb, D., Albertson, J. G., Salder, L, & Close, N. (2018). Minding the Baby: Complex trauma and attachment-based home intervention. In H. Steele & M. Steele (Eds). *Handbook of attachment-based interventions* (pp.151-174). The Guilford Press.

Somech, L.Y., & Elizur, Y. (2012) Promoting self-regulation and cooperation in pre-kindergarten children with conduct problems: A randomized controlled trial. *Journal of American Academy of Child and Adolescent Psychiatry, 51* (4):412-422. <https://pubmed.ncbi.nlm.nih.gov/22449647/>

Sourander, A., McGrath, P. J., Ristkari, T., Cunningham, C., Huttunen, J., Lingley-Pottie, P., Hinkka-Yli-Salomäki, S., Kinnunen, M., Vuorio, J., Sinokki, A., Fossum, S., & Unruh, A. (2016). Internet-assisted parent training intervention for disruptive behavior in 4-year-old children: A randomized clinical trial. *JAMA Psychiatry, 73*(4) 378-387. <https://doi.org/10.1001/jamapsychiatry.2015.3411>

**Appendix B**

Final Search Terms for Systematic Review

**Pubmed**

((((((child[mh] OR child[tw] OR children[tw] OR adolescent[mh] OR infant[mh] OR boy[tw] OR boys[tw] OR girl[tw] OR girls[tw]) AND (parent-child relations[mh:noexp] OR mother-child relations[mh:noexp] OR father-child relations[mh] OR parent[mh] OR parent[tw] OR parenting[tw] OR paternal[tw] OR maternal[tw] OR caregiver[tw] OR caregivers [tw] OR carer[tw] OR family[tw] OR families[tw]))) AND (emotion coaching[tw] OR emotion socialiali*[tiab] OR emotion-regulation[tw] OR emotion-focused[tw] OR emotion-based[tw] OR attachment*[tw] OR maternal sensitivity[tw] OR parental sensitivity[tw] OR reflective-function*[tiab] OR non-behavio*[tiab] OR nonbehavio*[tiab] OR video feedback[tw])) AND (Intervention[tw] OR prevention[tw] OR treatment[tw] OR therapy[tw] OR training[tw] OR program*[tiab])) AND (externalising[tw] OR externalizing[tw] OR psychopathology[mh] OR problem behavior[tw] OR attention deficit and disruptive behavior disorders[tw] OR oppositional[tw] OR conduct behavio*[tiab] OR conduct problem*[tiab]OR antisocial behavio*[tiab] OR prosocial behavio*[tiab] OR delinquen*[tiab] OR non-complian*[tiab] OR noncomplian*[tiab] OR aggressi*[tiab] OR child behavio*[tiab] OR internalising[tw] OR internalizing[tw] OR affective symptom[mh] OR anxiety disorders[mh] OR depressive disorder[mh] OR depression[mh])) AND (trial[tw] OR effect*[tiab] OR treatment outcome[mh:noexp] OR random*[tiab] OR influence[tw] OR predict[tiab])

**Cochrane**

**Population**

child OR adolescent OR infant OR teen OR teenager OR toddler OR youth OR “young person” OR “young people” OR juvenile OR boy OR girl

Parent OR mother OR father OR paternal OR maternal OR caregiver OR family OR families OR carer

**Intervention**

attachment or reflective-function or reflective-functioning or non-behavioural or non-behavioral or nonbehavioural or nonbehavioral OR emotion-regulation OR emotion-based OR emotion-focused OR sensitive-responding OR maternal-sensitivity OR parental-sensitivity OR emotion-coaching OR emotion-socialization OR video-feedback

**Comparator**

Intervention OR treatment OR training OR program* OR prevention OR therapy

**Outcome**

externalizing OR psychopathology OR internalizing OR aggressi*

(conduct OR antisocial OR prosocial OR non-compliance OR non-compliant OR noncomplian* OR aggressi* OR child OR affective OR depressi*) near/1 (behavior OR symptom OR disorder OR problem)

Problem Behavior; attention deficit and disruptive behavior disorders”; anxiety disorder OR depression; affective symptom

**Scopus**

((TITLE-ABS-KEY(child OR children OR adolescent OR infant OR boy OR girl OR juvenile OR teen OR teenager OR youth OR "young person" OR "young people" ) AND TITLE-ABS-KEY(parent OR mother OR father OR carer OR caregiver OR family OR families ) AND TITLE-ABS-KEY(intervention OR prevention OR treatment OR therapy OR program ) AND TITLE-ABS-KEY(trial OR effect* OR "treatment outcome" OR random* OR influence OR predict* ))) AND ((TITLE-ABS-KEY(attachment OR "maternal sensitivity" OR "parental sensitivity" OR "sensitive responding" OR "video feedback" OR "non behavioural" OR "non behavioral" OR nonbehavio*) OR TITLE-ABS-KEY((emotion OR emotional ) PRE/1 ( coaching OR sociali?ation OR focused OR based ) ))) AND ((TITLE-ABS-KEY(externali*ing OR psychopathology OR aggressi* OR noncompliance OR "non compliance" internali*ing OR defian*) OR TITLE-ABS-KEY((problem OR disruptive OR oppositional OR "non compliant" OR conduct OR antisocial OR prosocial OR child OR behavior OR anxiety OR depressi* OR affective ) PRE/1 ( disorder OR problem OR symptom OR behavior))))

**Psychinfo**

**Population**

Child or children or adolescent or infant or boy or girl or juvenile or teen or teenager or youth

AND

Parent or mother or father carer or caregiver or family OR families

**Intervention**
(attachment or "maternal-sensitivity" or "parental-sensitivity" or "sensitive-responding" or "video-feedback" or "non-behavioural" or "non-behavioral" or nonbehavio*

OR

(emotion or emotional) adj1 (coaching or sociali?ation or focused or based)

**Comparator**

(intervention or prevention or treatment or therapy or program)

**Outcome**

((disruptive or oppositional or "non compliant" or "noncompliant" or conduct or defiant or antisocial or prosocial or child or anxiety or depressi* or affective) adj1 (disorder or problem or symptom or behavior))

OR

(externali$ing or psychopathology or aggressi* or noncompliance or "non-compliance internali$ing"

OR
(behavio$r adj1 problem)

*Note.* Search terms were identified based on PICOS according to PRISMA 2009 checklist. For PsychInfo and Cochrane each category was combined with ‘AND.’

**Appendix C**

References of Included Studies

Adkins, T., Reisz, S., Hasdemir, D., & Fonagy, P. (2021). Family Minds: A randomized controlled trial of a group intervention to improve foster parents' reflective functioning. *Development and Psychopathology*, 1-15. <https://doi.org/10.1017/S095457942000214X>

Baker, M, Biringen, Z, Meyer-Parsons, B, Schneider, A. (2015). Emotional Attachment and Emotional Availability tele-intervention for adoptive families. *Infant Mental Health Journal, 36*(2), 179-192. <https://doi.org/10.1002/imhj.21498>

Becker-Weidman, A. (2006). Treatment for children with trauma-attachment disorders: Dyadic Developmental Psychotherapy. *Child and Adolescent Social Work Journal, 23*(2), 147-171. <https://doi.org/10.1007/s10560-005-0039-0>

Bernstein, R. E., Timmons, A. C., & Lieberman, A. F. (2019). Interpersonal violence, maternal perception of infant emotion, and Child-Parent Psychotherapy. *Journal of Family Violence, 34*, 309-320. <https://doi.org/10.1007/s10896-019-00041-7>

Carnes-Holt, K. & Bratton, S. C. (2014). The efficacy of Child Parent Relationship Therapy for adopted children with attachment disorders. *Journal of Counselling and Development, 92*(3)*,* 328-337. <https://doi.org/10.1002/j.1556-6676.2014.00160.x>

Cassidy, J. Brett, B. E., Gross, J. T., Stern, J. A., Martin, D. R, Mohr, J. J., & Woodhouse, S. S. (2017). Circle of Security-Parenting: A randomized controlled trial in Head Start. *Development and Psychopathology, 29*(2), 651-673. <https://doi.org/10.1017/S0954579417000244>

Diamond, G. S., Kobak, R. R., Krauthamer Ewing E. S., Levy, S. A., Herres, J. L., Russon J. M, & Gallop, R.J. (2019). A randomized controlled trial: Attachment-based family and nondirective supportive treatments for youth who are suicidal. *Journal of American Child and Adolescent Psychiatry, 58*(7), 721-731. <https://pubmed.ncbi.nlm.nih.gov/30768418/>

Diamond, G. S., Reis, B. F., Diamond, G. M., Siqueland, L, & Isaacs, L. (2002). Attachment-Based Family Therapy for depressed adolescents: A treatment development study. *Journal of the American Academy of Child and Adolescent Psychiatry, 41*(10), 1190-1196. <https://doi.org/10.1097/00004583-200210000-00008>

Diamond, G. S., Wintersteen, M. B., Brown, G. K., Diamond, G. M., Gallop, R., Shelef, K., & Levy, S. (2010). Attachment-Based Family Therapy for adolescents with suicidal ideation: A randomized controlled trial. *Journal of the American Academy of Child and Adolescent Psychiatry*, *49*(2), 122-131. <https://doi.org/10.1016/j.jaac.2009.11.002>

Dozier, M., Peloso, E., Lindheim, O., Gordon, M. K., Manni, M., Sepulveda, S., Ackerman, J., Bernier, A., & Levine, S. (2006). Developing evidence-based interventions for foster children: An example of a randomized clinical trial with infants and toddlers*. Journal of Social Issues, 62*(4), 767-785. <https://doi.org/10.1111/j.1540-4560.2006.00486.x>

Duncombe, M. E., Havighurst, S. S., Kehoe, C. E., Holland, K. A., Frankling. E. J., Stargatt, R. (2016). Comparing an emotion-and a behavior-focused parenting program as part of a multsystemic intervention for child conduct problems. *Journal of Clinical Child and Adolescent Psychology, 45*(3), 320-334. <https://doi.org/10.1080/15374416.2014.963855>

Firk, C., Dahmen, B., Dempfle, A., Niessen, A., Baumann, C., Schwarte, R., Koslowski, J., Kelberlau, K., Konrad, K., & Herpertz-Dahlmann, B. (2020). A mother-child intervention program for adolescent mothers: Results from a randomized controlled trial (the TeeMo study). *Development and Psychopathology,* *33*(3), 992-1005. <https://doi.org/10.1017/S0954579420000280>

Giannotta, F., Ortega, E., & Stattin, H. (2013). An attachment parenting intervention to prevent adolescents' problem behaviors: A pilot study in Italy. *Child and Youth Care Forum, 42*(1), 71-85. <https://doi.org/10.1007/s10566-012-9189-3>

Guild, D. J., Alto, M. E., Handley, E. D., Rogosch, F., Cicchetti, D., Toth, S. L. (2021). Attachment and affect between mothers with depression and their children: Longitudinal outcomes of Child Parent Psychotherapy. *Research on Child and Adolescent Psychopathology, 49*(5), 563-577. <https://doi.org/10.1007/s10802-020-00681-0>

Havighurst, S. S., Kehoe, C. E., & Harley, A. E. (2015). Tuning in to Teens: Improving parental responses to anger and reducing youth externalizing behavior problems. *Journal of Adolescence, 42,* 148-158. <https://doi.org/10.1016/j.adolescence.2015.04.005>

Havighurst, S. S., Wilson, K. R., Harley, A. E., & Kehoe, C. E. (2019). Dads Tuning in to Kids: A randomized controlled trial of an emotion socialization parenting program for fathers. *Social Development, 28*(4), 979-997. <https://doi.org/10.1111/sode.12375>

Havighurst, S. S., Wilson, K. R., Harley, A. E., Kehoe, C. E., Efron, D., & Prior, M. R. (2013). "Tuning in to Kids": Reducing young children’s behavior problems using an emotion coaching parenting program. *Child Psychiatry and Human Development, 44*(2), 247-264. <https://doi.org/10.1007/s10578-012-0322-1>.

Havighurst, S. S., Wilson, K. R., Harley, A. E., & Prior, M. R. (2009). Tuning into Kids: An emotion-focused parenting program – Initial findings from a community trial. *Journal of Community Psychology, 37*(8), 1008-1023. <https://doi.org/10.1002/jcop.20345>

Havighurst, S. S., Wilson, K. R., Harley, A. E., Prior, M. R., & Kehoe, C. (2010). Tuning in to Kids: Improving emotion socialization practices in parents of preschool children - Findings from a community trial. *The Journal of Child Psychology and Psychiatry, 51*(12), 1342-1350. <https://doi.org/10.1111/j.1469-7610.2010.02303.x>

Högström, J., Olofsson, V., Özdemir, M., Enebrink, P., & Stattin, H. (2017). Two-year findings from a national effectiveness trial: Effectiveness of behavioral and non-behavioral parenting programs. *Journal of Abnormal Child Psychology, 45,* 527-542. <https://doi.org/10.1007/s10802-016-0178-0>

Israel, P. & Diamond, G. S. (2012). Feasibility of Attachment Based Family Therapy for depressed clinic-referred Norwegian adolescents. *Clinical Child Psychology and Psychiatry, 18*(3), 334-350. <https://doi.org/10.1177/1359104512455811>

Katz, L. F., Gurtovenko, K., Maliken, A., Stettler, N., Kawamura, J., & Fladeboe, K. (2020). An emotion coaching parenting intervention for families exposed to intimate partner violence. *Developmental Psychology, 56*(3), 638–651. <https://doi.org/10.1037/dev0000800>

Kehoe, C. E., Havighurst, S. S., Harley, A. E. (2014). Tuning in to Teens: Improving parent emotion socialization to reduce youth internalizing difficulties. *Social Development, 23*(2)*,* 413-431. <https://doi.org/10.1111/sode.12060>

Kehoe, C. E., Havighurst, S. S., & Harley, A. E. (2020). Tuning in to Teens: Investigating moderators of program effects and mechanisms of change of an emotion focused group parenting program. *Developmental Psychology, 56*(3), 623-637. <http://dx.doi.org/10.1037/dev0000875>

Lieberman, A. F., Ippen, C. G., & Van Horn, P. (2006). Child-Parent Psychotherapy: 6-month follow-up of a randomized controlled trial. *Journal of the American Academy of Child and Adolescent Psychiatry, 45*(8), 913-918. <https://doi.org/10.1097/01.chi.0000222784.03735.92>

Lieberman, A. F., Van Horn, P., & Ippen, C. G. (2005). Toward evidence-based treatment: child-parent psychotherapy with preschoolers exposed to marital violence. *Journal of the American Academy of Child and Adolescent Psychiatry, 44*(12), 1241-1248. <https://doi.org/10.1097/01.chi.0000181047.59702.58>

Lind, T., Bernard, K., Ross, E., & Dozier, M. (2014). Intervention effects on negative affect of CPS-referred children: Results of a randomized clinical trial. *Child Abuse and Neglect, 38*(9)*,* 1459-1467. <https://doi.org/10.1016/j.chiabu.2014.04.004>

Lind, T., Bernard, K., Yarger, H. A., & Dozier, M. (2019). Promoting compliance in children referred to child protective services: A randomized clinical trial. *Child Development, 91*(2), 563-576. <https://doi.org/10.1111/cdev.13207>

Lind, T., Raby, K. L., Goldstein, A., Bernard, K., Caron, E. B., Yarger, H. A., Wallin, A., & Dozier, M. (2020). Improving social–emotional competence in internationally adopted children with the Attachment and Biobehavioral Catch-Up Intervention. *Development and Psychopathology*, *33* (3), 957-969. <https://doi.org/10.1017/S0954579420000255>

Liu, S., Phu, T., Dominguez, A., Hurwich-Reiss, E., McGee, D., Watamura, S., Fisher, P. (2021). Improving caregiver self-efficacy and children's behavioral outcomes via a brief strength-based video coaching intervention: Results from a randomized controlled trial. Prevention Science. *Prevention Science.* <https://doi.org/10.1007/s11121-021-01251-6>

Meybodi, F. A., Mohammadkhani, P., Pourshahbaz, A., Dolatshahi, B., & Havighurst, S. (2017). Reducing children behavior problems: A pilot study of Tuning in to Kids in Iran. *Iranian Rehabilitation Journal, 15*(3), 269-276. <http://irj.uswr.ac.ir/article-1-733-en.html>

Meybodi, F. A., Mohammadkhani, P., Pourshahbaz, A., Dolatshahi, B., & Havighurst, S. (2019). Improving Parent emotion socialization practices: Piloting Tuning in to Kids in Iran for children with disruptive behavior problems. *Family Relations, 68*(5), 596-607. <https://doi.org/10.1111/fare.12387>

Moss, E., Dubois-Comtois, K., Cyr, C., Tarabulsy, G. M., St-Laurent, D., & Bernier, A. (2011). Efficacy of a home-visiting intervention aimed at improving maternal sensitivity, child attachment, and behavioral outcomes for maltreated children: A randomized control trial. *Development and Psychopathology, 23*(1), 195-210. <https://doi.org/10.1017/S0954579410000738>

Opiola, K, K., & Bratton, S. C. (2018). The efficacy of Child Parent Relationship Therapy for adoptive families: A replication study. *Journal of Counseling and Development, 96*(2), 155-166. <https://doi.org/10.1002/jcad.12189>

Ordway, M. R., Sadler, L. S., Dixon, J., Close, N., Mayes, L., & Slade, A. (2014). Lasting effects of an interdisciplinary home visiting program on child behavior: Preliminary follow-up results of a randomized trial. *Journal of Pediatric Nursing, 29*(1), 3-13. <https://doi.org/10.1016/j.pedn.2013.04.006>

Oxford, M. L., Marcenko, M., Fleming, C. B., Lohr, M. J., & Spieker, S. J. (2016). Promoting birth parents' relationships with their toddlers upon reunification: Results from Promoting First Relationships home visiting program. *Children and Youth Services Review, 61,* 109-116. <https://doi.org/10.1016/j.childyouth.2015.12.004>

Oxford, M. L., Spieker, S. J., Lohr, M. J., & Fleming, C. B. (2016). Promoting first relationships: Randomized trial of a 10-week home visiting program with families referred to child protective services. *Child Maltreatment, 2*(4), 267-277. https://doi.org/10.1177/1077559516668274

Ozturk, Y., Moretti, M., & Barone, L. (2019). Addressing parental stress and adolescents’ behavioral problems through an attachment-based program: An intervention study*. International Journal of Psychology and Psychological Therapy, 19*(1), 89-100. <https://www.ijpsy.com/volumen19/num1/509.html>

Pasalich, D. S., Fleming, C. B., Oxford, M. L., Zheng, Y., & Spieker, S. J. (2016). Can parenting intervention prevent cascading effects from placement instability to insecure attachment to externalizing problems in maltreated toddlers? *Child Maltreatment, 21*(3), 175-185. <https://doi.org/10.1177/1077559516656398>

Purvis, K. B., Razuri, E. B., Howard, A. R. H, Call, C. D., DeLuna, J. H., Hall, J. S., & Cross, D. R. (2015). Decrease in behavioral problems and trauma symptoms among at-risk adopted children following trauma-informed parent training intervention. *Journal of Child and Adolescent Trauma, 8*, 201-210. <https://doi.org/10.1007/s40653-015-0055-y>

Razuri, E. B., Howard, A. R. H., Parris, S. R., Call, C. D., DeLuna, J. H., Hall, J. S., Purvis, K. B., & Cross, D. R. (2016). Decrease in behavioral problems and trauma symptoms among at-risk adopted children following web-based trauma-informed parent training intervention. J*ournal of Evidence-Informed Social Work, 13*(2), 165-178. <https://doi.org/10.1080/23761407.2015.1014123>

Rezvan, S., Bahrami, F., Abedi, M., Macleod, C., Doost, H. T. N., & Ghasemi, V. (2013). A preliminary study on the effects of attachment-based intervention on pediatric obsessive-compulsive disorder. *International Journal of Preventative Medicine, 4*(1), 78-87. <https://www.ncbi.nlm.nih.gov/pmc/articles/PMC3570916/>

Rolock, N., Ocasio, K., White, K., Havighurst, S., Cho, Y., Fong, R., Marra, L., & Faulkner, M. (2021). Tuning in to Teens (TINT) with adoptive parents and guardians in the US: The replication phase of intervention research. *Journal of Public Child Welfare, 15*(1), 22-51. <https://doi.org/10.1080/15548732.2020.1846660>

Spieker, S. J., Oxford, M. L., Kelly, J. F., Nelson, E. M., & Fleming, C. B. (2012). Promoting First Relationships: Randomized trial of a relationship-based intervention for toddlers in child welfare. *Child Maltreatment, 17*(4)*,* 271-286. <https://doi.org/10.1177/1077559512458176>

Sprang, G. (2009). The efficacy of a relational treatment for maltreated children and their families. *Child and Adolescent Mental Health, 14*(2), 81-88. <https://doi.org/10.1111/j.1475-3588.2008.00499.x>

Stams, G. -J., M., Juffer, F., van IJzendoorn, M. H., & Hoksbergen, R. C. (2001). Attachment-based intervention in adoptive families in infancy and children's development at age 7: Two follow-up studies. *British Journal of Developmental Psychology, 19*(2), 159-180. <https://doi.org/10.1348/026151001166010>

Stattin, H., Enebrink, P., Özdemir, M., & Giannotta, F. (2015). A national evaluation of parenting programs in Sweden: The short-term effects using an RCT effectiveness design. *Journal of Consulting and Clinical Psychology, 83*(6), 1069-1084. <http://dx.doi.org/10.1037/a0039328>

Tobon, A. L., Condon, E., Sadler, L. S., Holland, M. L., Mayes, L. C., & Slade, A. (2020). School age effects of Minding the Baby- An attachment-based home-visiting intervention - On parenting and child behaviors. *Development and Psychopathology*. 1-13. <https://doi.org/10.1017/S0954579420000905>

Van Doesum, K. T. M., Riksen-Walraven, J. M., Hosman, C. M. H., & Hoefnagels, C. (2008). A randomized controlled trial of a home-visiting intervention aimed at preventing relationship problems in depressed mothers and their infants. *Child Development, 79*(3), 547-561. <https://doi.org/10.1111/j.1467-8624.2008.01142.x>

Velderman, M. K., Bakermans-Kranenburg, M. J., Juffer, F., van IJzendoorn, M. H, Mangelsdorf, S. C., & Zevalkink, J. (2006). Preventing preschool externalizing behavior problems through video-feedback intervention in infancy. *Infant Mental Health Journal, 27*(5), 466-493. <https://doi.org/10.1002/imhj.20104>

Waraan, L., Rognli, E. W., Czajkowski, N. O., Aalberg, M., & Mehlum, L. (2021). Effectiveness of Attachment-Based Family Therapy compared to treatment as usual for depressed adolescents in community mental health clinics*. Child and Adolescent Psychiatry Mental Health, 15*(8), 1-14. <https://doi.org/10.1186/s13034-021-00361-x>

Weihrauch, L., Schäfer, R., & Franz, M. (2014). Long-term efficacy of an attachment-based parental training program for single mothers and their children: a randomized controlled trial. *Journal of Public Health, 22,* 139-153. <https://doi.org/10.1007/s10389-013-0605-4>

Wilson, K. R., Havighurst, S. S., Harley, A. E. (2012). Tuning in to Kids: An effectiveness trial of a parenting program targeting emotion socialization of preschoolers*. Journal of Family Psychology, 26*(1), 56 -65. <https://doi.org/10.1037/a0026480>

Wilson, K. R., Havighurst, S. S., Kehoe, C., Harley, A. E. (2016), Dads Tuning in to Kids: Preliminary evaluation of a fathers' parenting program*. Family Relations, 65*(4), 535-549. <https://doi.org/10.1111/fare.12216>

**Appendix D**

Characteristics of Included Studies

| Study Details | | Participant Details | | | | | Intervention Details | | | | Comparator Details | | Outcome Details | | |
| --- | --- | --- | --- | --- | --- | --- | --- | --- | --- | --- | --- | --- | --- | --- | --- |
| Study  First Author (Year) | N | Child Age (yrs.) | % Girls | % Mothers | Caregiver | Population Sample | Intervention Name | Group /Individual | # Sessions (Session length) | Setting | # arms | Comparator Type | Outcome Measure  (Informant) | Follow-up Period | SMD (95% CI) |
| Diamond (2002) | 32 | 13-17 | 78 | - | Birth | Clinical | ABFT | Group | 12 (1-1.5) | - | 2 | WL | YSR – Externalizing  YSR – Internalizing  BDI (child self-report)  HAM – D  (clinician) | Post | -0.49 [-1.20, 0.21]  -0.55[-0.26], 0.15)  -0.75[-1.47, -0.03]*  81% no longer met criteria for MDD vs. 47% of control |
| Diamond (2010) | 66 | 12-17 | 83 | - | Birth | Clinical | ABFT | Group | 12 (1-1.5) | Hospital/Medical | 2 | Enhanced Usual Care | BDI-II  (child self-report) | Post  6 months | -0.41[-0.90,0.08]  -0.26[-0.75, 0.22] |
| Diamond (2019) | 129 | 12-18 | 74 | - | Birth | Clinical | ABFT | Group | 16 (-) | Hospital/Medical | 2 | Active family-enhanced nondirective supportive therapy  (FE-NST) | BDI-II  (child self-report) | Post | -0.04[-0.38,0.31] |
| Israel (2012) | 20 | 13-17 | 55 | - | - | Clinical | ABFT | Individual | 12-17 (-) | Hospital/Medical Setting | 2 | TAU | BDI-II  (child self-report)  HAM-D  (clinician) | Post | -0.75[-1.67,0.17]  -1.03[-1.99, -0.08]* |
| Waraan (2021) | 60 | 13-18 | 86.7 | 57 | Unclear (Likely birth) | Clinical | ABFT | Individual | 16 | Community | 2 | TAU | HAM-D  (clinician) | Post | 0.32[-0.19, 0.83] |
| Rezvan (2013) | 24 | 10-12 | 100 | 100 | Birth | Clinical | “Attachment-Based Intervention” | Individual | 8 (1) | Hospital/ Medical | 2 | WL | CY-BOCS  (clinician) | Post  1- month | -1.37[-2.27,-0.46]**  -2.29[-3.35, -1.22]** |
| Stams (2001) | 35 | 7 - 7 | 54 | 100 | Adoptive, Birth | Community | “Attachment-Based Intervention” | Individual | 3 (-) | Home | 2 | “Control Group” (non-active) | CBCL - Internalizing  (parent) | 6 years | -0.70[-1.39, -0.02 |
| Dozier (2006) | 60 | 0.30-3.28 | 50 | 93 | Foster | Community | ABC | Individual | 10 (1) | Home | 2 | Active (Developmental Education Group) | PDR or PDR/IT  (parent) | - | - |
| Lind (2014) | 117 | 10-13 | 47 | 93 | Birth | Maltreatment exposed | ABC | Individual | 10 (-) | Home | 2 | Active (Developmental Education Group) | ‘Emotion Expression’  (clinician) | Post | -* |
| Lind (2019) | 101 | 0.28 – 2.15 | 49 | 100 | Birth | Maltreatment exposed | ABC | Individual | 10 (-) | Home | 2 | Active (Developmental Education Group) | Child Compliance  (clinician) | Approx. 29 months | -0.52 [-0.92, -0.12]* |
| Lind (2020) | 131 | 2-5 | 59 | 96 | Adoptive | Maltreatment exposed | ABC | Individual | 10 (1) | Home | 2 | Active (Developmental Education Group) | BITSEA  (parent)  DB- DOS- examiner present  (clinician)  DB-DOS - examiner busy  DB-DOS – parent context | Post  2 years | -0.52 [-0.94, -0.11]*  -0.53 [-0.95, 0.11]*  -0.40 [-0.82, 0.25]  -0.38 [-0.80, 0.03] |
| Sprang (2009) | 53 | 0-5 | 49 |  | Foster/ Adoptive | Maltreatment exposed | ABC | Individual | 10 (-) | Home | 2 | Active (WL + Support) | CBCL – Externalizing  (parent)  CBCL – Internalizing  (parent)  PSI  (parent self-report) | Post | -1.77[-2.41, -1.33]**  -1.58[-2.20, -0.95]**  -3.47[-4.34, -2.60]** |
| Cassidy (2017) | 141 | 3.32 – 5.30 | 58 | 100 | Birth | Community | Circle of Security (CoS) | Group | 10 (1.5) | Community Centre | 2 | WL | CBCL – Externalizing  CBCL – Internalizing  (parent) | Within 2 months | -0.08 [-0.25, 0.41]  -0.17 [-0.50, 0.16] |
| Bernstein (2019)  (same sample – Lieberman, 2005; 2006) | 113 | 2-6 | 48 | 100 | Birth | Maltreatment exposed | CPP | Individual | 50 (1) | - | 2 | Active ( TAU + Case Management)  SCL-90-R | CBCL – Externalizing  CBCL – Internalizing  (parent) | Post | 0.11 [-0.30,0.51]  -0.17 [-0.57,0.29] |
| Guild (2021) | 135 | 9-10 | 47 | 100 | Birth | Other (Mothers with depression) | CPP | Individual | 30-75 (-) | Home | 3 | ‘Depressed Control’(TAU)  ‘Non-Depressed Control’ (not included) | SCIPD - Child Oppositionality/Defiance SCIPD – Child Withdrawal  (clinician) | Approx. 6 years post-intervention | 0.11 [-0.3, 0.51]  0.26 [-0.16, 0.68] |
| Carnes-Holt (2014) | 61 | 2.0 – 10.0 | - | 64 | Adoptive | Community | CPRT | Group | 10 (2) | Community Centre | 2 | WL | CBCL – Externalizing  (parent) | Post | 0.11 [-0.40, 0.61] |
| Opiola (2018) | 49 | 2-9 | 49 | 61 | Adoptive | Clinical | CPRT | Group | 10 (2) | Community Centre | 2 | TAU | CBCL- Total  (parent)  PSI  (parent self-report) | Post (within week) | -0.57 [-1.14, 0.00]  -0.73 [-1.31, -0.15]* |
| Giannotta (2013) | 147 | 11-14 | 50 | 100 | Birth | Community | Connect | Group | 10 (1) | - | 2 | WL | ECBI Intensity  (parent) | Post | 0.13 [-0.25, 0.51] |
| Högström (2017) (Same sample -Stattin, 2015) | 749 | 3-12 | 37 | 85 | Birth | Clinical | Connect | Group | 10 (1.5) | ‘Regular care setting’ | 5 | WL  Active (Comet, Cope, Incredible Years) | ECBI Intensity  (parent)  ECBI – Problem  CES-D  (parent self-report)  CGSQ  (parent self-report) | Post  2 years  Post  2 years  Post  2 years  Post  2 years | Connect vs. Waitlist = -0.42[-0.64, - 0.20]**  Connect vs. Active = -0.00[-0.18, 0.17]  Connect vs. active = -0.09 [-0.21, 0.03]  Connect vs. WL = 0.00 [-0.22, 0.22]  Connect vs. Active = 0.28 [0.11, 0.46]  0.08 [-0.08, 0.24]  Connect vs. Waitlist = -0.17 [-0.39, 0.05]  Connect vs. Active = 0.06 [-0.11, 0.24]  0.17 [0.02, 0.33]  Connect vs. Waitlist = 0.00 [-0.22, 0.22]  Connect vs. Active =0.38 [0.20, 0.56]**  0.15 [-0.00, 0.31] |
| Ozturk (2019) | 44 | -  M=15 (1.4) | 34 | 75 | Birth | Community | Connect | Group | 10 (1.5) | Community Centre | 2 | WL | SDQ – Externalizing  (parent)  SDQ- Internalizing  SIPA- Parent Domain  (parent self-report)  SIPA –  Adolescent Domain  SIPA – APR Domain | Post | -0.15[-0.75, 0.44]  -0.27[-0.86, 0.32]  0.48 [-0.12, 1.08]  -0.45[-1.05, 0.15]  -0.06 [-0.65, 0.53] |
| Baker (2015) | 15 | 2.67 – 3.83 | 40 | 80 | Adoptive | Community | Emotional Attachment and Emotional Availability | Individual | 7 (1) | Home | 2 | WL | CBCL – Total  (parent)  PSI  (parent self-report) | 2 weeks | -1.62[-2.84, -0.04]**  -0.28 [-0.30, 0.74] |
| Katz (2020) | 50 | 6-12 | 53 | 100 | Unclear (Likely Birth) | Maltreatment exposed | Emotion Coaching Parenting Intervention for Intimate Partner Violence- Exposed Families | Group | 12 (-) | Community Centre | 2 | WL | CDI  (child self-report) | Post | -0.83 [-1.42, -0.25]** |
| Becker-Weidman (2006) | 64 | 5.30 – 16.20 | 41 |  | Adoptive/ Foster | Clinical | Dyadic Developmental Psychotherapy | Individual | ~23 (2) | Outpatient | 2 | TAU | CBCL – Aggressive Behaviors  (parent)  CBCL – Anxious/ Depressed | 1.1 years | -1.78 [-2.36, -1.19]**  -0.21 [-0.71, 0.28] |
| Adkins (2021) | 89 | 0.08-17.5 | 56.7 | 69 | Foster | Maltreatment exposed | Family Minds | Group | 4-6 (3) | Unclear | 2 | 4hr Foster Training Class | SDQ – Conduct  (parent)  SDQ- Emotion  PSI-SF -Difficult Child  (parent self-report) | Post  6-months | 0.10[-0.44, 0.65]  0.15[-0.40, 0.69]  -0.12[-0.68, 0.48]  - |
| Liu (2021) | 91 | 0.33 - 3 | 41.8 | 98.9 | Unclear (likely birth) | Maltreatment exposed | FIND | Individual | 10 | Home | 2 | TAU | CBCL/1.5-5/ BITSEA -Externalizing  (parent)  CBCL/1.5-5/ BITSEA –  Internalizing | Post | -0.23 [-0.65, 0.19]  0.13 [-0.29, 0.585] |
| Moss (2011) | 67 | 1.0 – 5.9 | 39 | 94 | Birth | Maltreatment exposed | Home Visiting | Individual | 8 (1.5) | Home | 2 | ‘Control – Monthly Visit from Child welfare caseworker “Standard Agency Care” | CBCL – Externalizing  (parent)  CBCL – Internalizing | Post (approx. within 2 weeks) | 0.03[-0.48, 0.54]  -0.12 [-0.63, 0.39] |
| Ordway (2014) | 50 | 3-5 | 44 | 100 | Birth | Community | MTB | Individual | 24+ (-) | Home | 2 | Routine Health Care | CBCL – Externalizing  (parent)  CBCL – Internalizing  CES-D (parent self-report) | 1 to 3 years | -0.89[-1.48, -0.31]  -0.35 [-0.91, 0.21]  0.50 [-0.06, 1.06] |
| Tobon (2020) | 97 | 4-10 | 47 | 100 | Unclear (Likely Birth) | Maltreatment exposed | MTB | Individual | 78 (-) | Home | 2 | TAU | CBCL – Externalizing  (parent)  CBCL – Internalizing | 2 to 8 years | -0.38 [-0.79, 0.02]  -0.32 [-0.72, 0.09] |
| Van Doesum (2008) | 71 | 0.43 – 1.85 | 39 | 100 | Birth | Other (parents with depression) | MTB | Individual | 8-10 (1-1.5) | Home | 2 | Telephone Calls | ITSEA – Externalizing  ITSEA - Internalizing  BDI  (parent self-report) | 6 months  Post  Follow-up | 0.09[-0.38, 0.55]  -0.12[-0.59, 0.34]  0.17 [-0.29, 0.62]  0.18[-0.27, 0.64] |
| Weihrauch (2014) | 58 | - | - | 100 | Birth | Community | PALME | Group | 20 (1.5) | Community Centre | 2 | WL | SDQ total (teacher report)  SCL-90-R- General Severity Index  (parent self-report) | Post  6 months  Post  6 months | -0.21 [-0.73, 0.31]  -0.27 [-0.79, 0.25]  -0.35 [-0.87, 0.17]  0.13[-0.39, -0.65] |
| Spieker (2012)  (Same sample - Oxford, 2016; Pasalich, 2016) | 210 | 0.8 - 2 | 44 | - | Birth Foster, & Kinship | Community | Promoting First Relationships | Individual | 10 (1-1.25) | Home | 2 | Education Support | CBCL – Externalizing  (parent)  CBCL – Internalizing  PSI-SF  (parent self-report) | 6 months  Post  6 months | -0.13[-0.47, 0.22]  -0.03[-0.38, 0.32]  0.17[-0.13, 0.46]  -0.05[-0.39, 0.30] |
| Firk (2020) | 56 | 0.25-0.5 | 48 | 100 | Birth | Other (adolescent mothers) | Steps Towards Effective and Enjoyable Parenting (STEEP-b) | Individual | 12-18 | Home | 2 | Publicly funded health care | BITSEA  (parent)  PSI – Total Scale  (parent self-report) | Post  6 months Post | 0.07[-0.51, 0.64]  -0.01[-0.59, 0.58]  -0.53 [--1.12, 0.05] |
| Purvis (2015) | 96 | -  M = 7.88 | 38 | 97 | Adoptive | Maltreatment exposed | TBRI | Group | 4 (6) | - | 2 | WL | SDQ – Conduct Problems  SDQ – Emotional Problems  (parent) | 2 weeks | -0.18[-0.58, 0.22]  -0.34 [-0.74, 0.07] |
| Razuri (2016) | 256 | -  M ~8 | 50 | - | Adoptive | Maltreatment exposed | TBRI | Individual | 18 (0.5) | Online | 2 | WL | SDQ – Conduct Problems  SDQ – Emotional Problems  (parent) | 2 weeks | 0.07[-0.18, 0.31]  -0.07 [ -0.31, 0.18] |
| Duncombe (2016) | 320 | 4.9-9.9 | 26 | 93 | - | Clinical | TIK | Group | 8 (2) | - | 3 | WL  Triple P Positive Parenting Program | ECBI Intensity (parent)  SDQ Conduct Problems (teacher) | 6 months | TIK vs. Waitlist = -0.25 [-0.53, 0.04]  TIK vs. Triple P = 0.10 [-0.17, 0.38]  TIK vs. Waitlist = -0.17 [-0.28, -0.06]**  TIK vs. Triple P = -0.05 [-0.32, 0.23] |
| Havighurst (2010)  (same sample – Havighurst, 2009) | 216 | 3.83-5.67 | 48 | 96 |  | Community | TIK | Group | 6 (2)  + 2 Booster sessions | Community Centre | 2 | WL | ECBI intensity  (parent)  ECBI  (teacher) | Post  6 months  6 months | -0.40[-0.70, -0.10]  -0.34 [-0.64, -0.04]*  -0.25 [-0.57, 0.06] |
| Havighurst (2013) | 54 | 4.00-5.92 | 76 | 100 |  | Clinical | TIK | Group | 6 (2) + 2 Booster sessions | Hospital/Medical Setting | 2 | TAU/WL “Usual Pediatric Care” | ECBI Intensity  (parent)  ECBI Problem  (parent)  ECBI Intensity (teacher)  ECBI Problem (teacher) | Post  6 months  Post  6 months | -0.59[-1.24, 0.07]  -0.00 [-0.62, 0.62]  -0.20 [-0.87, 0.46]  -0.07 [-0.71, 0.57]  -0.80 [-1.58, -0.01]  - 0.76 [-1.54, 0.02] |
| Meybodi (2019)  (Same sample - Meybodi, 2017) | 54 | 3-6 | 41 | 100 | Birth | Clinical | TIK | Group | 6 (2) + 2 booster sessions | - | 2 | WL | ECBI Problem  (parent)  ECBI Intensity  (parent)  GHQ-28  (parent self-report) | Post  3 months  Post  3 months  Post  3 months | -0.55 [-1.13, 0.03]  -0.87 [-1.47, -0.28]**  -0.09[-0.65, 0.48]  -0.18 [ -0.75, 0.39]  0.14[-0.43, 0.71]  -0.1[-0.57, 0.56] |
| Wilson (2012) | 128 | 4.0-5.11 | 50 | 92 | Birth | Community | TIK | Group | 6 (2) | Community Centre | 2 | WL | ECBI Intensity  (parent) | 7 months | -0.32[-0.67, 0.03] |
| Havighurst (2019) (Same sample - Wilson, 2016) | 162 | 3.05 – 6.34 | 46 | 0 | Birth | Community | Dads TIK | Group | 6 (2) + 2 booster sessions | Community Centre | 2 | WL | SDQ – Externalizing  SDQ – Internalizing  (parent) | Post  6 months  Post  6 months | -0.08[-0.39, 0.24]  -0.22 [-0.55, 0.10]  -0.41 [-0.73, -0.09]*  -0.41 [-0.73, 0.09]* |
| Kehoe (2020)  (Same sample - Havighurst,2015; Kehoe, 2014) | 225 | 10-13 | 51 | 89 | Birth | Community | TINT | Group | 6 (2) | Community Centre | 2 | WL | SDQ Externalizing  (parent)  SCAS  (parent)  SCAS (child self-report)  SDQ Externalizing (child self-report)  GHQ-28  (parent self-report) | 10-12 months post-baseline | -0.17[-0.44, 0.10]  -0.37[-0.63, -0.11]**  -0.33 [-0.60, -0.06]*  -0.24[-0.51, 0.33]  -0.18 [-0.45, 0.09] |
| Rolock (2021) | 769 | 10-13 | 49 |  | Adoptive & Kinship | Maltreatment-Exposed | TINT | Group | 7 (2) | Community Centre | 2 | TAU | BPI  (parent) | 6-8 months post-intervention | 0.08 [-0.10, 0.26] |
| Velderman (2006) | 77 | 0.58 – 0.83 | 53 | 100 | - | Community | Video-Feedback | Individual | 4 (1.5) | Home | 3 | Active (VIPP-R  ‘Control (No Intervention)’ | CBCL – Externalizing  CBCL- Internalizing  (parent) | 3.25 years | -0.33 [-0.88, 0.21]  0.09 [-0.45, 0.63] |

*Note.* ABFT = Attachment-Based Family Therapy; ABC = Attachment- and Biobehavioral Catch-up; APR = Adolescent-Parent Relationship; BPI = Behavioral Problem Index; BITSEA = Brief Infant Toddler Social Emotional Assessment; CBCL = Child Behavior Checklist; CDI = The Child Depression Inventory; CES-D = Center of Epidemiological Studies – Depression Scale; CGSQ = Caregiver Strain Questionnaire; CPP = Child Parent Psychotherapy ;CPRT = Child Parent Relationship Therapy; CY-BOCS = Children’s Yale-Brown Obsessive Compulsive Scale; DB-DOS = Disruptive Behavior Diagnostic Observation Schedule; ECBI = Eisenberg Child Behavior Inventory; FIND = Filming Interactions to Nurture Development; HAM-D = Hamilton Depression Rating Scale; HADS = Hospital Anxiety and Depression Scale; HIWC = Home Interview with Child; GHQ-28 = The General Health Questionnaire; MDD = Major Depressive Disorder; MTB = Minding the Baby; PDR/ IT= Parent Daily Report/ Infant-Toddler; PSI= Parenting Stress Index; PSI- SF= Parenting Stress Index – Short Form ; SCL-90-R = The Symptom Checklist-90-Revised; SCIPD = System for Coding Interactions in Dyads; SIPA = Stress Index for Parents of Adolescents; SDQ = Strengths and Difficulties Questionnaire; TAU = Treatment as Usual; TBRI = Trauma-Based Relationship Intervention; TIK = Tuning in to Kids; TINT = Tuning in to Teens; YSR = Youth Self-Report from Strengths and Difficulties Questionnaire; VIPP-R = Video-Feedback intervention to Promote Positive Parenting with a Representational Focus; WL = Waitlist.

**p < 0.05*

***p <0.01*

**Appendix E**

Risk of Bias of Studies included in Review

| **Study ID** | **Random Sequence Generation** | **Allocation Concealment** | **Blinding of Participants and personnel** | **Blinding of outcome Assessment** | **Incomplete Outcome Data** | **Selective Reporting** | **Fidelity** | **Other** | **Overall Risk of Bias** |
| --- | --- | --- | --- | --- | --- | --- | --- | --- | --- |
| **Adkins 2021** | Low | Low | High | High | Low | High | High | Low | High |
| **Baker 2015** | Unclear | Unclear | High Risk | Unclear | Low Risk | Low Risk | Low Risk | Unclear | Unclear |
| **Becker-Weidman 2006** | Unclear | Unclear | Unclear | Unclear | Unclear | Low | High | High | High |
| **Bernstein 2019** | Low | Unclear | Unclear | Unclear | Low | Low | Unclear | Low | Unclear |
| **Carnes-Holt 2014** | Low | Unclear | Low | Low | Low | Low | Low | Low | Low |
| **Cassidy 2017** | Unclear | Unclear | Unclear | Low | Low | Low | Low | Low | Unclear |
| **Diamond 2002** | Low | Unclear | Unclear | Low | Low | Low | Low | Low | Low |
| **Diamond 2010** | Low | Low | Unclear | Unclear | Low | Low | Unclear | Unclear | Unclear |
| **Diamond 2019** | Low | Low | Low | Low | Low | Low | Low | Low | Low |
| **Dozier 2006** | Low | Low | Low | Low | Low | Low | Low | Unclear | Unclear |
| **Duncombe 2016** | Unclear | Unclear | Low | Low | Low | Low | Low | Low | Low |
| **Firk 2020** | Low | Low | Low | Low | Unclear | Low | Unclear | Low | Unclear |
| **Gianotta 2013** | Unclear | High | Unclear | Unclear | Low | Low | Unclear | Low | High |
| **Guild 2021** | Low | Unclear | Unclear | Low | Low | Unclear | Unclear | Unclear | Unclear |
| **Havighurst 2010** | Low | Low | Unclear | Low | Low | Low | Low | Low | Low |
| **Havighurst 2012** | Low | Unclear | Unclear | Low | Low | Low | Low | Low | Low |
| **Havighurst 2019** | Unclear | Unclear | Unclear | Unclear | Low | Low | Low | Low | Unclear |
| **Hogstrom 2017** | Low | Low | Low | Unclear | Low | Low | Low | Low | Low |
| **Israel 2012** | Low | Low | Unclear | Low | Low | Low | Unclear | Unclear | Unclear |
| **Katz 2020** | Unclear | High | High | Unclear | Unclear | Unclear | High | Unclear | High |
| **Kehoe 2020** | Low | Low | Unclear | Low | Low | Low | Low | Low | Low |
| **Lind 2014** | Low | Low | Low | Low | Low | Low | Unclear | Low | Low |
| **Lind 2019** | Low | Unclear | Unclear | Low | Low | Unclear | Unclear | Low | Unclear |
| **Lind 2020** | Low | Low | Unclear | Unclear | Low | Unclear | Unclear | Unclear | Unclear |
| **Liu 2021** | Unclear | High | High | Unclear | High | Unclear | Low | Unclear | High |
| **Meybodi 2019** | Low | Unclear | Unclear | Unclear | Low | Low | Unclear | Unclear | Unclear |
| **Moss 2011** | Low | Low | Unclear | Low | Low | Low | Unclear | Low | Low |
| **Opiola 2018** | Low | Unclear | Unclear | Low | Low | Low | Low | Low | Low |
| **Ordway 2014** | Unclear | Unclear | Unclear | Low | Unclear | Low | High | Low | High |
| **Ozturk 2019** | Low | Unclear | Unclear | Unclear | Low | Low | Low | Unclear | Unclear |
| **Purvis 2015** | Unclear | Unclear | Unclear | Unclear | High | High | Unclear | Unclear | High |
| **Razuri 2016** | Low | Unclear | Unclear | Unclear | High | Unclear | Low | Unclear | High |
| **Rezvan 2013** | Low | Unclear | Unclear | Unclear | Low | Unclear | High | Unclear | High |
| **Rolock 2021** | Unclear | Unclear | Low | Unclear | Low | Unclear | Unclear | Unclear | Unclear |
| **Spieker 2012** | Low | Unclear | Unclear | Low | Low | Low | Low | Low | Low |
| **Sprang 2009** | Low | Unclear | Unclear | Low | Low | Low | Low | Unclear | Unclear |
| **Stams 2001** | Low | Unclear | Unclear | Low | Unclear | High | High | High | High |
| **Tobon 2020** | Unclear | Unclear | Unclear | Unclear | Low | Unclear | Unclear | Low | Unclear |
| **Van Doesum 2008** | Low | Low | Unclear | Low | Low | Low | Unclear | Unclear | Unclear |
| **Velderman 2006** | Unclear | Unclear | Unclear | Low | Low | Low | Unclear | Low | Unclear |
| **Waraan 2021** | Low | Low | Unclear | Low | High | High | Unclear | Low | High |
| **Weihrauch 2014** | Unclear | Unclear | Unclear | Unclear | High | Low | Unclear | High | High |
| **Wilson 2012** | Low | Low | Unclear | Unclear | Low | Low | Low | Low | Low |

**Appendix F**

Table of Attachment- and Emotion-Focused Parenting Interventions Considered to Target Child Mental Health Outcomes

| Targets Mental Health Outcomes | Does not Target Mental Health Outcomes |
| --- | --- |
| Attachment-Based Family Therapy (Diamond et al., 2002; Diamond et al., 2010; Diamond et al., 2019; Israel and Diamond, 2012; Waraan et al., 2021) | “Attachment-based intervention in adoptive families” (Stams et al., 2010) |
| Attachment-based intervention for pediatric obsessive-compulsive disorder (Rezvan et al., 2013) | Attachment and Bio-Behavioral Catch-up (ABC)  (Dozier et al., 2011); Lind et al, 2013, 2019, 2020; Sprang, 2009) |
| Child Parent Relationship Therapy (CPRT) (Carnes-Holt & Bratton, 2014; Opiola & Bratton, 2018) | Circle of Security (Cassidy et al. 2017) |
| Connect (Gianotta et al., 2013; Högström et al., 2017; Ozturk et al., 2019). | Child Parent Psychotherapy  (Bernstein et al., 2019; Guild et al., 2021) |
| Emotion Coaching Parenting Intervention for Intimate Partner Violence- Exposed Families (Katz et al., 2020) | Dyadic Developmental Psychotherapy (Becker-Weidman et al., 2006) |
| Promoting First Relationships (PFR) (Spieker et al, 2012) | Emotional attachment and emotional availability tele-intervention for adoptive families (Baker et al. 2015) |
| Tuning in to Kids (Duncombe et al., 2016; Havighurst et al., 2010, 2013, 2019; Kehoe et al., 2020; Meybodi et al., 2019; Wilson, 2012) | Family Minds (Adkins et al., 2021) |
| Tuning in to Teens (Havighurst et al., 2015; Kehoe et al., 2014; 2020; Rolock et al., 2021) | Filming Interactions to Nurture Development (FIND, Liu et al., 2021) |
| Dads Tuning in to Kids (Havighurst et al., 2019) | Home-Visiting Program (Moss et al., 2011) |
|  | Minding the Baby (Ordway et al., 2014; Tobon et al., 2020; Van Doesum, 2008) |
|  | PALME (Weihrauch et al., 2014) |
|  | Step Towards Effective and Enjoyable Parenting Brief (STEEP-b) (Firk et al., 2020) |
|  | Trauma-Based Relationship Intervention (TBRI)  (Purvis et al., 2015; Razuri et al., 2016) |
|  | “Video-Feedback” (Velderman et al., 2006) |

**Appendix G**

Meta-Analyses of Studies Examining Effects of Attachment- and Emotion-Focused Parenting Interventions on Reducing Child Externalizing and Internalizing Outcomes: Overall Findings Including Subgroup and Sensitivity Analyses

| Outcome | Details |  | K | SMD | 95%CI | I^2^ |
| --- | --- | --- | --- | --- | --- | --- |
| Externalizing |  |  |  |  |  |  |
|  | Waitlist Controls |  | 15 | -0.17 | -0.27, -0.06 | 26 |
|  |  | 0 to 5 | 6 | -0.19 | -0.35, -0.03 | 13 |
|  |  | 6 to 12 | 6 | -0.17 | -0.34, 0.00 | 49 |
|  |  | 13 to 17 | 3 | -0.07 | -0.41, 0.27 | 19 |
|  |  | Birth parents | 9 | -0.17 | -0.31, -0.03 | 28 |
|  |  | Non-birth parents | 3 | 0.02 | -0.18, 0.21 | 0 |
|  |  | Excluding high risk of bias | 12 | -0.23 | -0.33, -0.13 | 3 |
|  |  | Low risk only | 7 | -0.30 | -0.42, -0.18 | 0 |
|  |  | Immediate post only | 11 | -0.13 | -0.27, -0.02 | 42 |
|  |  | Follow-up only (6 months +) | 7 | -0.26 | -0.38, -0.13 | 0 |
|  |  | Clinical sample | 4 | -0.34 | -0.50, -0.18 | 0 |
|  |  | Community sample | 9 | -0.14 | -0.27, -0.01 | 12 |
|  |  | Designed to target child mental health | 11 | -0.23 | -0.34, -0.11 | 12 |
|  |  | Not designed to target child mental health | 4 | -0.01 | -0.18, 0.16 | 0 |
|  |  | Baseline clinically elevated | 7 | -0.20 | -0.34, -0.05 | 32 |
|  |  | Baseline not elevated | 7 | -0.17 | -0.34, -0.00 | 19 |
|  | Active Comparators |  | 18 | -0.30 | -0.51, -0.10 | 80* |
|  |  | Without Outliers | 16 | -0.13 | -0.26, -0.00 | 48 |
|  |  | 0 to 5 | 11 | -0.36 | -0.64, -0.09 | 74* |
|  |  | 6 to 12 | 6 | -0.27 | -0.59, 0.06 | 87* |
|  |  | 0 to 5 (without outlier) | 10 | -0.24 | -0.44, -0.04 | 46 |
|  |  | 6 to 12 (without outlier) | 5 | -0.00 | -0.13, 0.13 | 21 |
|  |  | Birth Parents | 9 | -0.06 | -0.20, 0.08 | 24 |
|  |  | Excluding high risk of bias | 13 | -0.09 | -0.22, 0.03 | 41 |
|  |  | Low risk of bias only | 5 | -0.02 | -0.15, 0.11 | 2 |
|  |  | Immediate post-only | 9 | -0.27 | -0.57, 0.03 | 77* |
|  |  | Follow-up (6 months +) | 13 | -0.28 | -0.48, -0.07 | 78* |
|  |  | Immediate Post-Only without outliers | 8 | -0.10 | -0.27, 0.07 | 27 |
|  |  | Follow-up (6 months +) without outliers | 12 | -0.16 | -0.31, -0.01 | 54 |
|  |  | Maltreatment-exposed sample | 8 | -0.16 | -0.37, 0.04 | 57 |
|  |  |  |  |  |  |  |
| Internalizing |  |  |  |  |  |  |
|  | Waitlist Controls |  | 11 | -0.34 | -0.51, -0.17 | 42 |
|  |  | Without outlier | 10 | -0.30 | -0.44, -0.15 | 23 |
|  |  | 0 to 5 | 3 | -0.22 | -0.48, 0.03 | 27 |
|  |  | Birth Parents | 6 | -0.38 | -0.57, -0.19 | 22 |
|  |  | Non-Birth Parents | 3 | -0.26 | -0.57, 0.05 | 44 |
|  |  | Excluding high risk of bias | 6 | -0.30 | -0.46, -0.15 | 0 |
|  |  | Immediate post only | 8 | -0.38 | -0.59, -0.15 | 48 |
|  |  | Follow-up (6 months +) | 4 | -0.35 | -0.56, -0.13 | 19 |
|  |  | Community sample | 6 | -0.31 | -0.46, -0.15 | 0 |
|  |  | Designed to target child mental health | 6 | -0.49 | -0.71, -0.28 | 23 |
|  |  | Not designed to target child mental health | 5 | -0.17 | -0.35, -0.01 | 11 |
|  |  | Baseline clinically elevated | 6 | -0.30 | -0.56, -0.05 | 55 |
|  |  | Baseline not elevated | 4 | -0.37 | -0.61, -0.13 | 25 |
|  |  |  |  |  |  |  |
|  | Active Comparators |  | 15 | -0.17 | -0.36, 0.02 | 60* |
|  |  | Without outlier | 14 | -0.08 | -0.20, 0.05 | 1 |
|  |  | Birth parents | 7 | -0.15 | -0.39, 0.08 | 49 |
|  |  | Excluding high risk of bias studies and outlier | 9 | -0.11 | -0.25, 0.04 | 1 |
|  |  | Low risk of bias only | 3 | -0.02 | -0.24, 0.20 | 0 |
|  |  | Immediate post only | 9 | -0.22 | -0.54, 0.09 | 73* |
|  |  | Follow-up (6 months +) | 6 | -0.10 | -0.45, 0.09 | 55 |
|  |  | Immediate post only without outliers | 5 | -0.05 | -0.23, 0.12 | 11 |
|  |  | Clinical Sample | 5 | -0.14 | -0.42, 0.14 | 37 |
|  |  | Maltreatment-exposed sample | 6 | -0.29 | -0.70, 0.13 | 78* |
|  |  | Maltreatment-exposed sample (without outlier) | 5 | -0.09 | -0.29, 0.11 | 0 |
|  |  | Designed to target child mental health | 5 | -0.09 | -0.35, 0.17 | 36 |
|  |  | Not designed to target child mental health | 10 | -0.20 | -0.47, 0.06 | 68* |
|  |  | Not designed to target child mental health (without outlier) | 9 | -0.08 | -0.23, 0.08 | 0 |

*Note.* If there were less than three studies available to conduct a subgroup analysis/sensitivity analysis it was not carried out and will not appear in this table.

*Heterogeneity considered too high, interpret with caution.
